# Novel approaches for long-term lung transplant survival

**DOI:** 10.3389/fimmu.2022.931251

**Published:** 2022-07-27

**Authors:** Cynthia L. Miller, Jane M. O, James S. Allan, Joren C. Madsen

**Affiliations:** ^1^Center for Transplantation Sciences, Massachusetts General Hospital, Boston, MA, United States; ^2^Division of Thoracic Surgery, Department of Surgery, Massachusetts General Hospital, Boston, MA, United States; ^3^Division of Cardiac Surgery, Department of Surgery, Massachusetts General Hospital, Boston, MA, United States

**Keywords:** lung transplantation, acute cellular rejection (ACR), chronic lung allograft dysfunction (CLAD), immunosuppression, *ex vivo* lung perfusion (EVLP), tissue-resident memory T-cells, mesenchymal stromal cells (MSCs), antibody-mediated rejection (AMR)

## Abstract

Allograft failure remains a major barrier in the field of lung transplantation and results primarily from acute and chronic rejection. To date, standard-of-care immunosuppressive regimens have proven unsuccessful in achieving acceptable long-term graft and patient survival. Recent insights into the unique immunologic properties of lung allografts provide an opportunity to develop more effective immunosuppressive strategies. Here we describe advances in our understanding of the mechanisms driving lung allograft rejection and highlight recent progress in the development of novel, lung-specific strategies aimed at promoting long-term allograft survival, including tolerance.

## Introduction

Lung transplantation has evolved significantly since its introduction in 1963 and is now commonly performed for a variety of end-stage lung diseases. Despite this, survival after lung transplant remains poor and has not significantly improved over the past several decades. Standard-of-care immunosuppressive regimens utilized in lung transplantation have failed to achieve acceptable long-term graft and patient survival. The median 6.7-year post-transplant survival represents one of the lowest among solid organs and is limited primarily by allograft failure due to acute and chronic rejection (1, 2). Recent insights into the unique immunologic properties of lung allografts have provided a framework to better understand the limitations of conventional immunosuppression in lung transplantation and provide an opportunity to develop novel strategies that take advantage of these properties (3).

**Figure 1:**
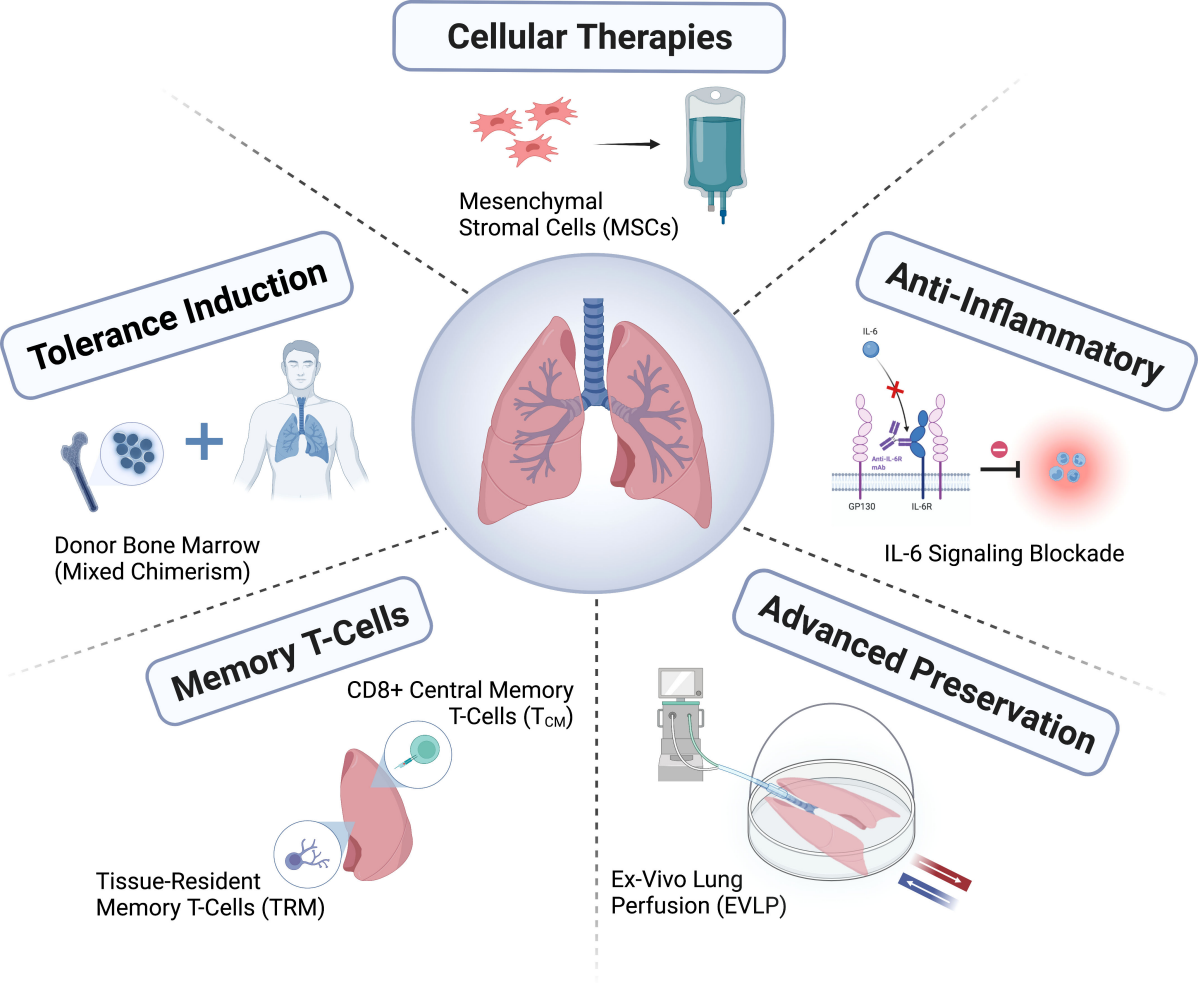
Novel approaches to achieve long-term lung transplant survival.

In this review, we describe recent advances in our understanding of the mechanisms driving lung allograft rejection, particularly in the context of limitations related to conventional immunosuppression. We then highlight existing and emerging lung-specific strategies aimed at promoting long-term allograft survival. Specifically, we describe novel preservation methods, cellular therapies, anti-inflammatory agents, strategies targeting memory T-cells, and tolerance induction (**Figure 1**).

## Mechanisms of lung allograft failure

### Acute inflammation

Beginning immediately after transplantation, lung allografts are at risk of primary graft dysfunction (PGD), a form of acute lung injury that can result in severe intra-graft inflammation. It is widely accepted that PGD, which occurs in up to 25% of lung transplants (4), is mediated by ischemia reperfusion injury (IRI) and represents an independent risk factor for the subsequent development of chronic allograft lung dysfunction (CLAD) (5–7).

### Adaptive immunity

Adaptive immune activation resulting in acute cellular and antibody-mediated rejection contribute significantly to early graft failure, with acute cellular rejection (ACR) occurring in 30-50% of recipients in the first year after transplantation (8, 9). Numerous studies have supported a role for ACR in subsequent development of CLAD, with increased risk associated with greater frequency and histologic severity of ACR (10–15). Antibody-mediated rejection (AMR) has emerged as one of the most vexing challenges in lung transplantation and is characterized by allograft dysfunction, the presence of circulating donor-specific antibodies (DSA), capillary endothelial C4d deposition and pathological findings characteristic of acute lung injury (16). This condition often leads to acute graft failure and can also predispose to the development of CLAD (17).

### Chronic lung allograft dysfunction

CLAD occurs in up to half of recipients within five years of transplantation and represents the principal life-limiting factor for lung transplant patients (18). The development of CLAD is likely multifactorial and related to the complex interaction of immune and non-immune factors, including but not limited to acute rejection, pre-transplant allosensitization, bacterial infection and colonization, acute viral infection, and gastroesophageal reflux disease; in a subset of patients, no clear risk factors for CLAD are identified and it is presumed to result from chronic rejection (19–22). CLAD is marked by fibrotic remodeling within the pulmonary allograft, with described phenotypes include bronchiolitis obliterans syndrome (BOS), restrictive allograft syndrome (RAS), and a mixed BOS-RAS phenotype (23). Histologic changes of BOS include dense submucosal fibrosis in membranous and respiratory bronchioles resulting in luminal occlusion and vasculopathy, whereas RAS is characterized by fibrosis of the alveolar interstitium, visceral pleura, and interlobular septa (14, 24).

### Unique immunobiology of lung allografts

Lung allografts possess a number of distinctive features that contribute to poor outcomes including 1) a large total surface area of vascular endothelium, 2) constant exposure to environmental antigens, and 3) an abundance of lymphoid tissue patrolled by a robust innate immune system. The expansive vascular endothelium of lung allografts results in increased susceptibility to ischemia reperfusion and complement-mediated injury, leading to an influx of neutrophils/macrophages into the graft with early T-cell activation and consequent rejection (25, 26). Due its role as a barrier organ, the lung is continually exposed to environmental antigens resulting in stimulation of toll-like receptors (TLRs) which activate proinflammatory innate and adaptive immune responses, thereby promoting rejection (27). Moreover, there exists abundant intragraft lymphoid tissue containing resident monocytes that can interact with and prime T-cells within the lung itself (28). Other important properties of lung allografts include the existence of immunomodulatory tissue-resident memory T-cells (TRMs) and CD8+ memory T-cells shown to promote allograft tolerance (29–33).

Similar to lung allografts, the skin harbors tissue-resident immune cells (including T-effector memory cells) and is continually exposed to the environment. As with the lung, the presence of local T-effector memory cells enables a more potent response to alloantigen than would occur following activation of naïve T cells. When compared to solid organ transplantation, recipients of vascularized composite allografts experience a much higher incidence of acute rejection (85% during the first year after transplantation) (34).

It is increasingly recognized that immune pathways driving lung allograft rejection and tolerance differ from those of other solid organs, likely due to the unique immunobiology of lung allografts (3, 28–31). One primary difference is regulation of alloimmune responses at the level of the lung allograft, which is in contrast with other transplanted organs that depend on cell trafficking to secondary lymphoid organs for activation of allorecognition pathways (28–30, 35–37). Early graft injury resulting from IRI and infection has been shown to activate innate immune pathways within the lung allograft, which in turn trigger alloantigen-specific T-cell expansion (19, 25, 38). During IRI, resident donor monocytes in the lung elaborate chemotactic and proinflammatory cytokines that facilitate neutrophil entry into lungs grafts, enhancing CD4^+^ T-cell responses to donor antigens and resulting in PGD (39–41). Additionally, lung monocytes can interact with Th17 cells and contribute to the development of CLAD (42).

The design of conventional immunosuppression has focused on controlling T-cell mediated responses. However, it is increasingly clear that inflammation, along with humoral and innate immunity, also play critical roles in lung allograft survival. As such, it is not surprising that current immunosuppressive regimens are unsuccessful in achieving acceptable long-term graft and patient survival. Moreover, conventional immunosuppression fails to account for the unique properties of lung allografts and as a result may negatively impact tolerogenic cell populations while allowing for the propagation of immune pathways leading to rejection. Taken together, these findings highlight the need for novel, lung-specific therapies that promote long-term allograft and patient survival.

## Novel preservation methods

Ischemia reperfusion injury is a critical mediator of PGD, which affects up to 25% of lung transplant recipients and is associated with the development of CLAD and late mortality (4, 43–45). Novel perseveration strategies aimed at reducing the incidence of IRI and subsequent PGD are critical to promoting long-term graft and patient survival. Moreover, the shortage of acceptable lung allografts calls for innovative methods that enable expansion of the donor organ pool.

### *Ex-vivo* lung perfusion

Conventional cold static preservation is performed by flushing donor lungs with a specialized preservation solution followed by hypothermic storage on ice (~4°C). The goal of hypothermic storage is to sustain cellular viability by reducing cellular metabolism, with maximum accepted preservation times limited to <8 hours with cold static preservation (46). During this process, a lack of arterial blood supply results in anaerobic metabolism, failure of ion-exchange channels, cell swelling, and impaired enzymatic activity (47). Upon donor lung reperfusion, oxidative stress from mitochondrial damage and electrolyte imbalance promotes local inflammation and results in release of reactive oxygen species, pro-inflammatory cytokines, proteases, and expression of damage-associated molecular patterns (DAMPs) (48–52). Consequent activation of the innate immune system further contributes to the inflammatory cascade and promotes harmful adaptive immune responses driven by alloreactive T-cells (53, 54). The culmination of these events results in the tissue damage that characterizes IRI.

EVLP involves machine preservation of donor lungs in a perfused, ventilated, normothermic condition, thereby reducing tissue injury resulting from anaerobic metabolism and hypothermia (55). EVLP represents a novel method for the assessment and treatment of donor lungs, with the ultimate goal of expanding the donor organ pool and improving long-term outcomes. Since first used clinically in 1991 (56), EVLP has undergone system modifications primarily relating to technical parameters of the platform (static *vs*. portable) and composition of the perfusate (cellular *vs*. acellular). Other proposed modifications involve donor lung positioning (supine *vs*. prone) (57), mode of ventilation (positive *vs*. negative-pressure) (58), and perfusate temperature (normothermic *vs*. subnormothermic) (59, 60). Currently, three EVLP platforms are approved for clinical use and include the Organ Care System Lung (OCS, Transmedics, Andover, MA), Vivoline LS1 (Vivoline Medical, Lund, Sweden), and the XPS XVIVO Perfusion AB system (XVIVO Perfusion, Gothenburg, Sweden). The major difference between these platforms is that they are either mobile, enabling donor lungs to be placed on EVLP immediately after procurement, or fixed, requiring transport of donor lungs to a specialized perfusion center.

EVLP offers several advantages over cold static preservation, including 1) the opportunity for evaluation of marginal donor lungs prior to transplantation, 2) safe extension of preservation time, and 3) the potential to improve both marginal and standard grafts through targeted administration of therapeutics (61). EVLP provides insight into the function of a marginal donor organ by enabling assessment of physiological parameters including pulmonary vascular pressures, perfusate oxygen content (PaO2), lung edema, and PaO2:FiO2 ratios, as well as novel parameters such as interstitial fluid metabolite composition (62). Extended duration of donor lung preservation through use of EVLP offers the advantage of greater flexibility in timing of graft implantation, reduction in the physical limitations related to organ allocation, and enables performance of advanced diagnostics and therapeutics that require longer periods of perfusion (63). EVLP using the Toronto Protocol has been shown to safely maintain lungs for >12 hours (46), with extended preservation to 24 hours using perfusate modifications that reduce metabolite accumulation and electrolyte imbalances known to occur during prolonged EVLP (64).

Perhaps the most significant advantage of EVLP relates to its use as a therapeutic platform for administration of localized, targeted lung-specific therapies (61, 65, 66). Even without modifications, EVLP is associated with alterations in the donor lung environment from diminished release of inflammatory mediators and augmentation of anti-inflammatory signaling pathways (67–70). EVLP has been shown to alter the inflammatory signaling profile of the donor lung, with a global profile of cellular survival and anti-apoptotic signature (67). Experimental studies have investigated administration of agents aimed at augmenting the anti-inflammatory properties of EVLP, many of which have shown promise in preventing inflammatory cytokine release and reactive oxygen species (ROS) generation during IRI. Such agents include alpha1-anti-trypsin (71), adenosine A1A-receptor agonists (72, 73) and A2B-receptor antagonists (74), K (ATP) channel modulators (75), ROS scavengers and PARP inhibitors (76–79). By removing donor leukocytes prior to transplantation, EVLP alters immunogenicity of the graft resulting in reduced allorecognition, T-cell priming, and T-cell infiltration in the recipient (68). EVLP has been used for targeted delivery of immunosuppressive agents including perfusate-based methylprednisolone (80), intrabronchial adenoviral human IL-10 (81), and drugs targeting leukocyte activation and function (82, 83). Further applications of EVLP involve modification of lung properties (84), anti-microbial treatment (85), and administration of cellular-based therapies (86–91).

Clinical trials have evaluated the impact of EVLP on outcomes in standard and extended criteria donors, as well as conversion rates for grafts initially deemed unsuitable for transplant. To date, EVLP has failed to demonstrate superior survival in clinical studies of standard criteria donor lungs (92, 93). The randomized INSPIRE trial evaluated EVLP outcomes with the OCS platform compared to traditional cold storage in standard criteria lungs, demonstrating a 50% reduction in rate of grade 3 PGD (PGD3) with EVLP but no statistically significant difference in short- and long-term survival (92). Similarly, a randomized trial using the XVIVO platform for standard criteria donors demonstrated no significant difference in 30-day survival compared to standard donor lung preservation, and similar short-term clinical outcomes between groups (duration of intubation, length of intensive care unit (ICU) and hospital stay) (93).

Remarkably, trials using EVLP for extended criteria donors have shown equivalent survival compared to standard criteria donors without EVLP (94, 95). The EXPAND trial applied the OCS system in extended criteria donors (donation after circulatory death (DCD), >age 55, PaO2:FiO2 ≤300, expected ischemic time >6 hours), with a 99% 30-day survival but a 44% rate of PGD3 at 72 hours (94). Additional studies of extended criteria donors using EVLP have shown similar clinical outcomes compared to standard criteria donors regarding rates of PGD3, duration of ICU and hospital stay, and 30-day or 1-year survival (95). Other prospective multicenter studies using the Vivoline perfusion system for extended criteria lungs initially declined for transplantation showed similar 1-year survival despite inferior short-term parameters (higher rate of extracorporeal membrane oxygenation (ECMO) support, duration of ICU stay/time to extubation) when compared to conventional donor lungs (96, 97). Importantly, in the prospective, multi-center NOVEL trial, lungs initially deemed unacceptable for transplant were screened using EVLP, resulting in a 50.9% conversion rate and equivalent short- and long-term outcomes compared to standard criteria lungs (98).

In sum, EVLP has been shown to reduce tissue inflammation and downregulate harmful immune responses, thereby improving the function of donor lungs. Current evidence from clinical trials supports the use of EVLP in extended criteria donors and as a mechanism to screen for viable grafts among the unused donor pool, enabling equivalent short- and long-term outcomes compared to standard criteria donor lungs. To date, there is a lack of demonstrated superiority using EVLP in clinical trials of standard criteria lungs. However, there exists great potential for further modifications to EVLP to enhance outcomes of both standard and marginal/extended criteria lungs with the overall effect of improved graft and patient survival.

### Xenogenic cross-circulation

Cross-circulation represents an advanced method of *ex vivo* lung preservation which, unlike machine perfusion, provides full physiologic support to donor lungs. In a porcine lung model, allogeneic cross-circulation with a host swine has been shown to successfully regenerate severely injured lungs and support lungs *ex vivo* for up to four days (99–101). Based on these results, the concept of xenogeneic cross-circulation of injured human lungs with living swine has been proposed with successful functional and histologic recovery of severely injured human lungs after 24 hours of xenogeneic cross-circulation (102). Cross-circulation with xenogeneic support may result in superior outcomes compared to isolated machine perfusion by providing systemic physiologic regulation in the *ex vivo* setting (103). However, the clinical applicability of xenogeneic cross-circulation as an approach for lung preservation and rehabilitation is limited by immunologic and ethical barriers as well as feasibility.

### Modifications to cold static preservation

Despite the advantages of *ex vivo* perfusion, considerable limitations exist including complexity and cost; as such, cold static storage remains the clinical standard of donor lung preservation. There have been few major changes in the technique of cold static preservation in the past decades, but several modifications have been proposed. One such modification involves use of a temperature-controlled preservation device, the Paragonix LUNGguard™ Donor Lung Preservation System (Paragonix, Braintree, Mass). This device enables continual monitoring of storage temperature and maintenance in the range of 4-8°C for prevention of freeze injury or inadvertent warming during donor lung transport and storage. Use of increased storage temperature of 10°C rather than 4°C during static preservation has also been proposed, with a recent study demonstrating improved mitochondrial health after prolonged preservation at 10°C compared to the standard 4°C (104). While the optimal lung preservation solution has yet to be established, Perfadex (XVIVO Perfusion AB, Gothenburg, Sweden) is a commonly used, low-potassium dextran solution. Recently, the use of a hydrogen-rich preservation solution comprised of dissolved hydrogen in Perfadex has been proposed as a mechanism to mitigate lung IRI by reducing levels of inflammatory cytokines, oxidative stress markers, and vascular endothelial dysfunction (105, 106).

## Cellular therapies

The use of cellular-based therapies in solid organ transplantation continues to evolve, with emerging roles for regulatory T-cells, B-cells, macrophages, dendritic cells, and genetically-modified CAR T-cells in prevention/treatment of IRI and rejection. The majority of such therapies have been explored in kidney and liver transplantation, however, mesenchymal stromal cells (MSCs), as well as epithelial progenitor cells (EPCs) and regulatory T-cells (Tregs) have shown promise in lung transplantation.

### Mesenchymal stromal cells

Mesenchymal stromal cells (MSCs, also seen as mesenchymal stem cells) are multipotent cells with a fibroblast-like morphology that were first isolated from bone marrow and spleen in 1970 (107). MSCs are a heterogenous population, but according to the International Society for Cellular Therapy Standards they must: 1) adhere to plastic, 2) be CD105+CD90+CD73+ and CD45-CD34-CD14-CD11b-CD79a-CD19-HLA-DR-, and 3) have the capacity to differentiate into the mesenchymal lineages (either bone, cartilage, or fat) (108). MSCs have been predominantly isolated from bone marrow, but umbilical cord MSCs and adipose-derived MSCs are also widely used.

Since their discovery, MSCs have also been found as resident cells in many organs throughout the body, including the lungs (109). In bone marrow, MSCs produce a host of trophic and regulatory factors to create a niche to support stem cell hematopoiesis (110), and it is thought that lung resident MSCs serve to similarly support bronchoalveolar stem cell populations and help direct the maintenance and repair of lung tissues (111).

MSCs also have several unique features that make them particularly attractive candidates for cell therapy in lung transplantation. First, they are either very long-lived or capable of self-renewal; in gender-mismatched lung transplantation, MSCs have been shown to retain a donor’s gender even years post-transplant (112). Second, they express little to no co-stimulation or MHC markers and are poorly immunogenic, to the extent they have been successfully transplanted across not only HLA barriers but also across species (113). This has led many groups to transition away from bespoke cultured MSCs and toward more shelf-stable allogeneic cell lines, often derived from neonatal stem cells, that do not require *de novo* MSC isolation and GMP culture. Third, MSCs have been found to be possess a potent immunomodulatory capacity, which may differ based on their source (114). *In vitro* studies show that they inhibit effector T-cell activation (115) and may promote regulatory T-cells over pro-inflammatory Th17 cells (114, 116). In the presence of inflammation as occurs in the setting of transplantation, MSCs may be primed to possess an even greater capacity for immunosuppression *via* TNF-TNFR2 dependent signaling (114, 117). MSCs secrete a variety of cytokines, chemokines, and inflammatory factors that regulate the immune system and have anti-oxidative and anti-apoptotic functions (118–122) (reviewed in (123, 124)). Fourth, because of their large size after *in vitro* culture, MSCs may become trapped in the lungs following intravenous infusion (125). The effect of MSCs can be seen throughout the body due to paracrine and secreted factors, but MSCs can also migrate to sites of tissue injury, and it is possible that lungs derive additional benefit from the presence of MSCs locally in the graft. Finally, MSCs have already been tested in human studies for a wide variety of indications including acute respiratory distress syndrome (ARDS), with doses ranging up to 100 million cells/kg without evidence of significant adverse effects (126–129).

### MSC-derived extracellular vesicles

Of note, some of the effects of MSCs do not require cell-cell contact *in vitro*, and MSCs have been found to exert effects on distant organs *in vivo*. It is thought that MSCs release not only paracrine signaling molecules, but also extracellular vesicles (EVs). EVs are small, membrane-bound, non-nucleated particles actively assembled and released by cells including MSCs. EVs can contain proteins, mRNA, and miRNA which can be transmitted to another cell. As the field evolves, guidelines have been proposed by the International Society for Extracellular Vesicles to more formally categorize these EVs by origin (whether plasma membrane or endosome), size, density, etc. (130). EVs are also being investigated as a means of inducing the beneficial effects of MSCs without the risk of alloreactivity to cells or the entrapment of cells in the lungs.

### Preclinical studies of MSCs in lung IRI

MSCs, MSC-derived EVs, and MSC-derived conditioned media (which contain EVs) have been evaluated in dozens of studies of lung IRI. Most studies used rodent models with IRI induced by hyperoxia, ventilator damage, E. coli bacteria/LPS, or chemicals such as bleomycin [reviewed in (123)]. Consistently, treatment with MSCs has been found to result in increased survival and decreased lung injury and edema.

Nearly all studies found that MSC-treated subjects had more favorable, anti-inflammatory cytokine and growth factor profiles. Neutrophil infiltration was decreased with MSC treatment (87, 131–135) while there was an increase in M2-like macrophages and regulatory T-cells (121, 136). The p38 MAPK is inhibited and Bcl-2 is translocated to the nucleus, preventing apoptosis (137). In other organ models, MSCs have been found to transfer mitochondria and glycolytic enzymes, ameliorating mitochondrial dysfunction that can lead to excessive ROS generation and metabolic dysfunction (138). After treatment with MSCs, autophagy was markedly decreased (139). In rodents with elastase-induced emphysema and bleomycin-induced fibrosis, both emphysema and fibrosis were decreased as well (136, 140).

### MSC delivery using EVLP

Most preclinical studies of MSC treatment for lung IRI use intravenous or intraperitoneal infusion to deliver the cells, however, studies in lung transplantation have the unique opportunity to use EVLP for cell delivery. Combining MSC treatment with EVLP may enable the targeting of IRI before it occurs and reconditioning of lungs without the theoretical risks of microvascular embolism from high dose intravenous infusion of MSCs [reviewed in (141) and (142)].

In one large animal transplant study, swine lungs were perfused with human umbilical cord MSCs after 24 hours of static cold storage and an additional 12 hours of EVLP. Once transplanted and reperfused, lungs treated with MSCs had significantly reduced acute lung injury scores, improved wet-to-dry ratios, lower levels of inflammatory cytokines, and higher levels of growth factors than the control group, suggesting an amelioration of IRI associated with the transplantation process (86). Previous studies from the same group confirmed that intravascular perfusion led to better retention of cells in the parenchyma than intrabronchial delivery of MSCs, and that larger numbers of cells perfused offered little benefit over the optimum dose of 5 million cells/kg (143, 144).

MSCs may also reduce the risk of pulmonary edema and PGD by increasing alveolar fluid clearance (AFC) rates, which can be negatively impacted by IRI-associated damage to the alveolar epithelium. A handful of studies have been published evaluating human lungs treated with MSCs or multipotent adult progenitor cells (MAPCs) *ex vivo*. After 4-12 hours of EVLP, IL-8, IL-10, TNFa, pulmonary vascular resistance (PVR), and oxygenation were not demonstrably changed in most studies. However, one study found that histologic lung injury scores were decreased, and studies that evaluated AFC consistently found that AFC was improved or restored to normal levels. Two additional studies have also evaluated microvesicles derived from bone marrow MSCs and similarly found that AFC rates were improved (**Table 1**).

**Table 1:** Studies of mesenchymal stromal cells (MSCs), multipotent adult progenitor cell (MAPCs), and MSC-derived extracellular vesicles (EVs) in human lung *ex vivo* lung perfusion (EVLP).

Lead Author	Year	Cells	Dose	Lung injury model	EVLP time	Outcome
Lee (143)	2009	BM MSC from NIH repository, Tulane Center for Gene therapy	5 million	<30h ischemic time plus E. coli bacteria/endotoxin	4h	AFC restored, IL-8, IL-10, TNFa unchanged
Lee (145)	2013	GMP BM MSC from University of Minnesota	5 million	<48h ischemic time plus endotoxin or E. coli bacteria	6-10h	AFC restored, IL-8 and TNFa decreased, IL-10 increased *in vitro*
McAuley (146)	2014	GMP BM MSC from University of Minnesota	5 million	>30h cold ischemia	4h	No change
La Francesca (147)	2014	MAPC	10 million	8h cold ischemia	4h	Reduced lung injury score, reduced neutrophils and eosinophils
Gennai (148)	2015	BM MSC-derived EV	MV from 10-20 million MSCs	Lungs rejected for transplant (<48h cold ischemia, no parenchymal lesions, and AFC >0% but <10%/h)	8h	Improved AFC, restored tracheal pressure, increased compliance relative to baseline, reduced PAP or PVR. No significant difference in oxygenation
Park (149)	2019	BM MSC-derived MV	MV from 20-40 million MSCs	E. coli bacteria	6h	Improved AFC, no change in PAP, PVR, compliance, or oxygenation
Nykanen (150)	2021	UC MSC modified to produce IL-10	40 million	<10 hours cold ischemia	12h	No difference in PVR, oxygenation, compliance, airway pressure

BM, Bone marrow; MSC, mesenchymal stromal cell; GMP, Good manufacturing process; AFC, Alveolar fluid clearance; MAPC, Multipotent adult progenitor cell; EV, Extracellular vesicle; MV, Microvesicle; PAP, Pulmonary artery pressure; PVR, Pulmonary vascular resistance; UC, Umbilical cord.

### MSC-based cellular therapy in human lung transplantation

Based on the results from early studies demonstrating the safety and feasibility of MSC administration in human lung transplant recipients (128, 129), there are currently three clinical trials of allogeneic MSC infusion for lung transplantation on clinicaltrials.gov (**Table 2**). Two of the trials aim to evaluate the effect of MSCs on lung transplant recipients with BOS or CLAD, while one aims to evaluate the effects of MSCs on PGD in all lung transplant recipients.

**Table 2:** Currently registered clinical trials of mesenchymal stromal cells (MSCs) in lung transplant recipients.

Lead institution	Type	Phase	Patients	MSC source	Intervention	Primary outcome	NCT	Status
Rigshospitalet, Denmark	Double blind	1/2	All lung transplant	Allogeneic adipose	100 million vs 200 million vs placebo	PGD	NCT04714801	Recruiting
Mayo clinic, MN	Non-randomized	1	BOS+	Allogeneic bone marrow	0.5 million vs 1 million +/- 1 million booster	Safety, changes in PFTs	NCT02181712	Completed 2021
University of Queensland, Australia	Randomized	2	CLAD+	Allogeneic bone marrow	8 million/kg vs placebo	Progression-free survival from CLAD	NCT02709343	Recruiting

PGD, Primary graft dysfunction; BOS, Bronchiolitis obliterans syndrome; PFT, Pulmonary function test; CLAD, Chronic lung allograft dysfunction.

Unresolved questions remain about the subtle differences between MSCs derived from different sources (i.e., bone marrow *vs*. umbilical cord blood), their performance and viability suspended in acellular perfusate compared to blood-based perfusate (as is used in normothermic machine perfusion) or culture media, and the possible toxic effects of dimethyl sulfoxide used as a cryoprotectant for frozen MSCs. Additionally, MSCs are a heterogeneous population and autologous cells can vary significantly between patients. The development of standardized allogeneic cell preparations can alleviate some of these concerns, as can the use of EVs instead of cells.

In sum, there exists mounting evidence demonstrating the beneficial effects of MSCs in treating lung injury in animal models and on *ex vivo* perfused human lungs. As a result, there exists great potential for use of MSC-based therapies to help expand the donor pool by reconditioning marginal lungs and by limiting or treating lung injury after transplantation.

### Endothelial progenitor cells

Endothelial injury represents a critical component in the pathogenesis of lung IRI, wherein release of proinflammatory cytokines from hypoxemia and reoxygenation results in compromised endothelial integrity and increased alveoli-capillary permeability. During reperfusion, overproduction of ROS and activation of cell adhesion molecules causes endothelial swelling and detachment from the basement membrane, resulting in increased vascular permeability that enables leukocyte entry into the tissues (151–153). Release of proinflammatory mediators by activated macrophages causes lung injury characterized by damage to pulmonary and alveolar endothelial cells (154–156). Strategies aimed at protecting the lung endothelium include inhibition of ROS, targeting of endothelial adhesion molecules, and endothelial glycolax stabilization (153).

More recently, the use of endothelial progenitor cells (EPCs) has been explored as a mechanism by which to attenuate lung injury and promote vascular regeneration. EPCs possess anti-inflammatory properties and a capacity for re-endothelialization; in models of acute lung injury, EPCs have been shown to preserve pulmonary endothelial function and prevent increased permeability of the pulmonary alveolar-capillary barrier (157, 158). The potential for EPCs in attenuating lung IRI has been demonstrated in animal models of lung transplantation, in which administration of autologous EPCs improved lung allograft survival and function in the setting of prolonged ischemia (159). Additional findings support the ability for EPCs to ameliorate lung IRI through downregulation of inflammatory and endothelial adhesion molecule expression *via* the endothelial NOS (eNOS) pathway (160). Further research will help to elucidate the role for administration of EPCs as a therapeutic strategy to mitigate lung IRI.

### Regulatory T-cells

Tregs are known to mediate alloimmunity are known to mediate alloimmunity in solid organ transplantation and play a distinct role in lung transplantation due to local immunoregulation within the allograft. The formation of bronchus-associated lymphoid tissue (BALT), a tertiary lymphoid organ enriched in T_regs_, is associated with the development of lung transplant tolerance (30). T_regs_ not only participate in local mechanisms of tolerance induction within the lung allograft, but can also regulate peripheral immune responses *via* lymphatic egress (161). Further support for the tolerogenic role of graft resident T_regs_ is evidenced by development of AMR after selective depletion of intragraft T_regs_ from tolerant lung allografts (162). In human lung transplant recipients, reduced percentage of T_regs_ in BAL fluid correlates with rejection (163), and increased levels of circulating T_regs_ are associated with improved graft survival (164, 165).

Only recently have T_regs_ been investigated as a cellular therapy in lung transplantation. Pre-transplant administration of *in-vitro* expanded recipient T_regs_ using EVLP was performed in a porcine model of lung transplantation and discarded human lungs (166); T_regs_ were shown to enter the lung parenchyma in both models and retain suppressive function, and thereby represent a promising strategy for local immune regulation prior to transplantation. However, the administered T_regs_ only remained in the graft for 3 days after transplantation with no significant difference in acute rejection between the control and T_regs_ -treated groups by day 7, suggesting that lung allograft residency may be required for T_regs_ to exert their tolerogenic effects.

## Anti-inflammatory strategies

### IL-6 signaling blockade

IL-6 is a pleiotropic, pro-inflammatory cytokine that has been linked to lung allograft inflammation and immune -mediated graft injury, with elevated IL-6 levels shown to be predictive of PGD and 30-day mortality (167–169). Evidence from multiple model systems mechanistically link IL-6 to the inflammatory cascades inherent to IRI and downstream dendritic cell and T-cell activation/infiltration that result in graft rejection (28, 54, 170, 171). Moreover, studies of PGD in lung transplantation support a role for IRI-induced proinflammatory gene expression including IL-6 (167, 169). Monocytes/macrophages including alveolar macrophages express the IL-6 receptor (IL-6R) and generate pro-inflammatory responses comprised of inflammasome activation, production of toxic reactive oxygen and nitrogen species, and release of cytokines including IL-6, culminating in acute and chronic allograft immunopathology (172, 173). Thus, initiation of agents aimed at IL-6 signaling inhibition prior to reperfusion in lung transplantation offers the potential to limit innate inflammation downstream of IRI and thereby reduce the development of PGD.

The potential benefits of IL-6 signaling inhibitors in lung transplantation surpass their anti-inflammatory effects, given the role of IL-6 in the adaptive immune responses responsible for development of acute and chronic lung transplant rejection (174–178). Agents currently approved for clinical use or in development include primarily those that target IL-6 (clazakizumab, siltuximab) or the IL-6 receptor (tocilizumab, sarilumab) (179, 180). Our group previously achieved long-term lung allograft survival in non-human primates (NHPs) by supplementing conventional triple drug immunosuppression with anti-thymocyte globulin induction therapy and a short post-operative course of anti-IL6R therapy with tocilizumab (181). Recent clinical trials in kidney transplantation have focused on the use of IL-6 signaling blockade for desensitization and for the treatment of acute and chronic AMR, with promising initial results (182–187). In addition, a multicenter phase II clinical trial investigating the efficacy of tocilizumab in cardiac transplantation has been initiated (NCT03644667), with clinical trials in lung transplantation forthcoming.

### JAK inhibitors

The JAK/STAT pathway is essential for cytokine signaling involved in upregulation of acute inflammatory pathways, including those associated with lung allograft rejection. JAK-dependent cytokines including IFN gamma and IL-5 have been shown to be upregulated in CLAD (188), and elevated levels of CXCR3 chemokines downstream from JAK-dependent IFN gamma signaling have been associated with worse outcomes in lung transplant recipients (189). Use of systemic JAK inhibitors for prevention of allograft rejection has shown efficacy in kidney transplantation but was associated with increased rates of infection and posttransplant lymphoproliferative disease (190).

Recently, lung-specific, inhaled (non-systemic) JAK inhibitors have been developed and offer potential for use in the prevention of acute and chronic lung allograft rejection. The use of inhaled, lung-specific JAK inhibitors provides local drug delivery with minimal systemic effects and has shown efficacy in corticosteroid-resistant pulmonary inflammation (191). The inhaled pan-JAK inhibitor nezulcitinib (TD-0903, Theravance Biopharma) was used for treatment of COVID-19 associated lung injury in a Phase II clinical trial (NCT04402866) but failed to meet its primary endpoint; however, the drug was well-tolerated and showed a trend towards decreased mortality and duration of hospitalization (192).

### Azithromycin

Azithromycin is a macrolide antibiotic with diverse antibacterial, antiviral, and anti-inflammatory effects, and has been shown to attenuate airway and systemic inflammation after lung transplantation (193–195). A randomized-controlled trial demonstrated that use of prophylactic azithromycin combined with conventional immunosuppression improved post-transplant outcomes including lung allograft function and risk of CLAD; its efficacy was attributed to reduced airway and systemic inflammation based on lower C-reactive protein levels in patients receiving azithromycin (194). Although it has been shown to decrease the rate of CLAD and improve long-term survival after lung transplantation (196–198), post-transplant azithromycin did not result in improved early allograft function in a randomized-controlled trial (199). However, this study confirmed the known anti-inflammatory properties of azithromycin with lower bronchoalveolar lavage (BAL) neutrophilia and IL-8 levels at 30 and 90 days post-transplant (199).

### Perfusion-based strategies

The process of EVLP using normothermic machine perfusion with an acellular perfusate has been shown in experimental studies to mitigate lung allograft inflammation by downregulating the release of pro-inflammatory mediators and promoting anti-inflammatory pathways. Specifically, use of EVLP is associated with diminished release of DAMPs including mitochondrial DNA (mt-DNA) (67, 200) and decreased levels of pro-inflammatory cytokines such as IL-6, IL-1 beta, IL-18, and TNF-alpha (69, 70, 201). Correspondingly, EVLP induces the expression of genes encoding for anti-inflammatory pathways, including feedback inhibitors of TLRs and regulatory cytokines (i.e., IL-10) (69, 200).

Beyond its intrinsic anti-inflammatory effects, EVLP enables targeted delivery of anti-inflammatory agents as additives to the perfusate. In an experimental model, reconditioning of lung allografts with Cyclosporine A during EVLP attenuated proinflammatory changes and mitigated mitochondrial dysfunction, resulting in improved early graft function after transplantation (202). Administration of anti-inflammatory adenosine A2A-receptor agonists and pro-inflammatory A2B-receptor antagonists was also shown to improve lung quality and was associated with diminished expression of pro-inflammatory cytokines including CXCL1, CCL2, TNF-alpha, IFN gamma, and IL-12 (72–74, 203). Other perfusion-based therapeutics aimed at diminishing cell death and inflammation include ROS scavengers/PARP inhibitors and alpha1-anti-trypsin (71, 76, 79). Finally, implementation of a cytokine adsorber to the EVLP system for cytokine removal has shown promise in limiting the inflammatory response in experimental models (204).

## Tissue-resident and central memory T-cells

### Tissue-resident memory T-cells

Tissue-resident memory T-cells (TRMs) represent a unique population of non-circulating T-cells with memory features that serve a critical role in the defense against infectious pathogens at barrier surfaces (205, 206). TRMs are defined by their commitment to non-lymphoid peripheral tissues and lack of recirculation and are distinct from circulating central memory and effector memory T-cells (207–210). As a key immunological barrier organ, the lung is highly enriched for TRMs that have the potential to mediate rapid, *in-situ* immune responses (211, 212). TRMs therefore serve an important role in localized immunity with significant implications regarding allogeneic responses within lung allografts.

Donor-derived T-cells have been identified in lung allografts as TRMs for >1 year after transplantation, and their long-term persistence is associated with reduced incidence of PGD and ACR (32). In contrast, lung TRMs derived from infiltrating recipient T-cells gradually acquire TRM markers in the months after transplant and may mediate allograft rejection (32, 213, 214). Longitudinal analysis of lung transplant recipient BAL has demonstrated lower levels of donor CD4+ and CD8+ T-cell chimerism in recipients who developed PGD compared to those who did not (32). Similarly, BAL samples from recipients with ACR demonstrated lower levels of donor T-cell chimerism, indicating that infiltrating recipient T-cells may mediate development of ACR. Taken together, these findings suggest that future therapies aimed at maintaining donor-derived TRMs and preventing their replacement by recipient TRMs may improve post-transplant outcomes.

More recently, the existence of recirculating TRMs has been identified, implying that TRMs may be capable of participating in systemic recall responses (215–217). Furthermore, the observation that graft-infiltrating recipient T-cells gradually acquire TRM markers suggests that these cells may actually represent repopulation of donor lung tissue by circulating effector memory T-cells that then acquire TRM phenotypes or by recirculating ex-TRMs (218). The ability of TRM cells to influence the immune environment within lung allografts through rapid *in-situ* and potentially also systemic immune responses represents yet another avenue for targeted interventions to prevent allograft injury and rejection.

### CD8+ central memory T-cells

Memory T-cells are generally viewed as pathogenic in the context of solid organ transplantation, with early infiltration of CD8+ memory T-cells into allografts shown to result in accelerated rejection (219–223). CD8+ alloreactive memory T-cells generated through heterologous immunity are considered a barrier to long-term graft survival due to a relative resistance to traditional immunosuppression. As a result, lymphoablative strategies to globally deplete T lymphocytes or specifically eliminate memory T-cells have been developed.

However, there exists emerging evidence that under certain circumstances memory T-cells maintain a regulatory capacity and suppress deleterious pro-inflammatory immune responses (224). Recent findings in lung transplantation support a critical role of CD8^+^ central memory T-cells (T_CM_) in the induction of transplant tolerance (29, 31). In a murine model of lung transplantation, it was shown that CCR7-expressing CD8+ T_CM_ cells promote costimulatory blockade-mediated lung allograft acceptance *via* IFN gamma-dependent nitric oxide production. The induction of tolerance by CD8+ T_CM_ cells was dependent on CCR7 expression, which enabled prolonged and stable interaction with intragraft antigen-presenting cells resulting in IFN gamma production, induction of nitric oxide, and downregulation of immune responses (29). The tolerogenic effect of CD8+ T_CM_ cells was shown to be dependent on expression of programmed cell death 1 (PD-1) by the CD8+ T_CM_ cells; in the absence of PD-1, these cells instead differentiated into an effector memory phenotype with subsequent allograft rejection (31). These findings suggest that indiscriminate T-cell depletion or targeting of pathways critical to the function of tolerogenic CD8+ T_CM_ cells may interfere with lung allograft acceptance and promote rejection. As such, a critical reevaluation of current immunosuppressive strategies is warranted to ensure optimization of protective cell populations in lung transplantation.

## Tolerance induction

**Figure 2:**
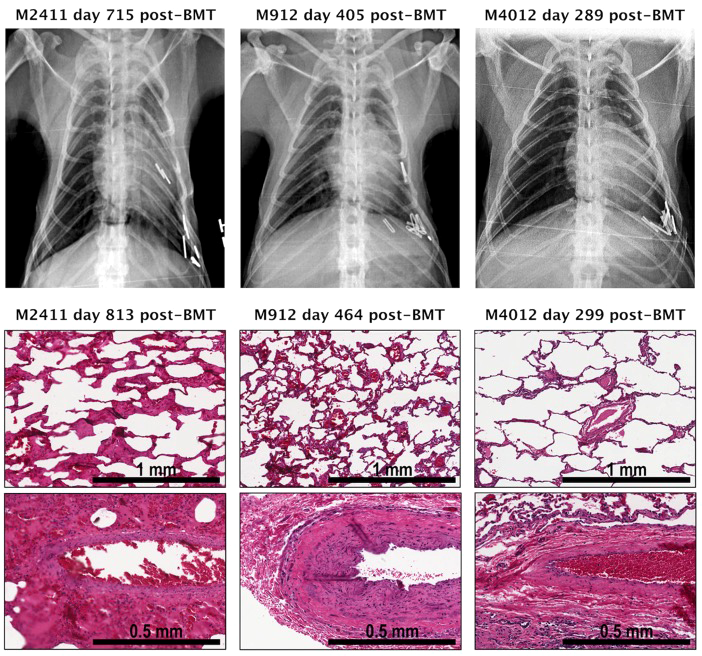
Pathology and chest radiographs from three tolerant NHP lung transplant recipients that underwent delayed donor bone marrow transplantation. Shown are photomicrographs (hematoxylin and eosin (H&E) staining) of lung biopsies performed at the time of euthanasia and chest radiographs obtained at indicated time points from each recipient. The chest radiographs of NHP recipients M2411, M912, M4012 displayed well-aerated lung allografts in the left thoracic space. No signs of rejection were seen in the lung graft biopsy of M2411, M912, and M4012. *Adapted from* (181). BMT, Bone marrow transplantation.

Induction of immune tolerance remains the ultimate goal in the field of transplantation, as it would enable indefinite graft survival in the absence of ongoing immunosuppression. In contrast to kidney and liver allografts, lung allografts are considered along with other “tolerance-resistant” organs due to their unique immune characteristics (225). While tolerance to kidney allografts has been achieved in human patients through mixed chimerism with donor bone marrow transplantation (226), such an approach has yet to reach the clinical realm for lung transplant recipients. Our group was the first to demonstrate the successful induction of lung allograft tolerance in a NHP model using a mixed chimerism strategy with anti-IL6R therapy (181). Four cynomolgus NHPs underwent MHC-mismatched lung transplantation followed by 4-month delayed donor bone marrow transplantation using a non-myeloablative mixed chimerism conditioning regimen (including anti-IL6R therapy). Three of the four NHP recipients achieved tolerance with long-term lung allograft survival off immunosuppression (**Table 3**); the tolerant NHPs had no evidence of acute or chronic allograft rejection, exhibited donor T-cell unresponsiveness, and did not develop donor-specific alloantibody (**Figure 2**). These findings represent significant progress towards clinical lung transplant tolerance, with tolerance induction representing yet another promising approach for improved outcomes following lung transplantation.

**Table 3:** Outcomes of four NHP lung transplant recipients that underwent delayed donor bone marrow transplantation for induction of lung allograft tolerance. *Adapted from* (181).

NHP Recipient	MHC Mismatch		Mixed Chimerism	ACR at last biopsy	Chronic rejection	Allo-antibody	Graft survival
	MHC I	MHC II
M4012	2/4	2/4	Permanent	ACR 0 Day 610 Post-LTx	None	None	299 days post-BMT (euthanized with no signs of rejection)
	Haploidentical				
M2411	2/4	2/4	Permanent	ACR 0 Day 939 Post-LTx	None	None	813 days post-BMT (euthanized with no signs of rejection)
	Haploidentical				
M912	2/4	2/4	Transient until Day 75 post-BMT	ACR 0 Post-mortem	None	None	464 days post-BMT (euthanized with no signs of rejection)
M4711	4/4	4/4	None	ACR 3 Post-mortem	Severe OB	Developed post-lung tx	176 days post-BMT

ACR, Acute cellular rejection; BMT, Bone marrow transplantation; LTx, lung transplant; OB, Obliterative bronchiolitis.

Moreover, long-term graft survival without the need for immunosuppression in the setting of tolerance induction would obviate the need for patient adherence to immunosuppressive regimens. Patient adherence remains a major barrier in achieving long-term survival following solid organ transplantation, including the lung (227–229). A recent meta-analysis evaluating the efficacy of interventions aimed at improving adherence identified the importance of a multidisciplinary team, a comprehensive intervention approach, and mobile health monitoring (230). Indeed, optimization of strategies that increase patient adherence represents an important aspect for improved outcomes following lung transplantation.

## Summary and conclusion

Although the field of lung transplantation has evolved significantly in the past several decades, there exists an urgent need for the development of more effective strategies to improve graft and patient outcomes. An improved understanding of the unique immunologic properties of the lung has helped to elucidate the mechanisms by which conventional immunosuppression fails to achieve long-term lung transplant survival. Promising future strategies include targeted delivery of lung-specific therapeutics using *ex vivo* lung perfusion, novel anti-inflammatory approaches (i.e., IL-6 signaling blockade), application of cell-based therapies (i.e., MSCs), harnessing of the regulatory potential of lung-specific memory T-cell populations, and tolerance induction. The ongoing development of advanced therapies in lung transplantation provides hope for a better future for lung transplant recipients.

## Author contributions

CM and JO performed the literature search and wrote the manuscript. JM and JA supervised, revised, and provided critical review of the manuscript. All authors contributed to the article and approved the submitted version.

## Funding

This work was supported by NIH grants P01HL018646, P01AI123086, U01AI131470, and R25AI147393 and the Nina Starr Braunwald Research Fellowship Award from The Thoracic Surgery Foundation to CM.

## Conflict of interest

The authors declare that the research was conducted in the absence of any commercial or financial relationships that could be construed as a potential conflict of interest.

## Publisher’s note

All claims expressed in this article are solely those of the authors and do not necessarily represent those of their affiliated organizations, or those of the publisher, the editors and the reviewers. Any product that may be evaluated in this article, or claim that may be made by its manufacturer, is not guaranteed or endorsed by the publisher.

## References

[1] Bos S Vos R Van Raemdonck DE Verleden GM. Survival in adult lung transplantation: Where are we in 2020? Curr Opin Organ Transplant (2020) 25(3):268–73. doi: 10.1097/MOT.0000000000000753 32332197

[2] Chung PA Dilling DF. Immunosuppressive strategies in lung transplantation. Ann Transl Med (2020) 8(6):409. doi: 10.21037/atm.2019.12.117 32355853 PMC7186623

[3] Shepherd HM Gauthier JM Kreisel D. Tolerance, immunosuppression, and immune modulation: Impacts on lung allograft survival. Curr Opin Organ Transplant (2021) 26(3):328–32. doi: 10.1097/MOT.0000000000000871 PMC8523032 33782247

[4] Lee JC Christie JD. Primary graft dysfunction. Proc Am Thorac Soc (2009) 6(1):39–46. doi: 10.1513/pats.200808-082GO 19131529

[5] Kreisel D Krupnick AS Puri V Guthrie TJ Trulock EP Meyers BF. Short- and long-term outcomes of 1000 adult lung transplant recipients at a single center. J Thorac Cardiovasc Surg (2011) 141(1):215–22. doi: 10.1016/j.jtcvs.2010.09.009 21093882

[6] Daud SA Yusen RD Meyers BF Chakinala MM Walter MJ Aloush AA. Impact of immediate primary lung allograft dysfunction on bronchiolitis obliterans syndrome. Am J Respir Crit Care Med (2007) 175(5):507–13. doi: 10.1164/rccm.200608-1079OC 17158279

[7] DerHovanessian A Weigt SS Palchevskiy V Shino MY Sayah DM Gregson AL. The role of tgf-beta in the association between primary graft dysfunction and bronchiolitis obliterans syndrome. Am J Transplant (2016) 16(2):640–9. doi: 10.1111/ajt.13475 PMC4946573 26461171

[8] Hachem RR Khalifah AP Chakinala MM Yusen RD Aloush AA Mohanakumar T. The significance of a single episode of minimal acute rejection after lung transplantation. Transplantation (2005) 80(10):1406–13. doi: 10.1097/01.tp.0000181161.60638.fa 16340783

[9] Todd JL Neely ML Kopetskie H Sever ML Kirchner J Frankel CW. Risk factors for acute rejection in the first year after lung transplant. A multicenter study. Am J Respir Crit Care Med (2020) 202(4):576–85. doi: 10.1164/rccm.201910-1915OC PMC7427399 32379979

[10] Girgis RE Tu I Berry GJ Reichenspurner H Valentine VG Conte JV. Risk factors for the development of obliterative bronchiolitis after lung transplantation. J Heart Lung Transplant (1996) 15(12):1200–8. 8981205

[11] Sharples LD McNeil K Stewart S Wallwork J. Risk factors for bronchiolitis obliterans: A systematic review of recent publications. J Heart Lung Transplant (2002) 21(2):271–81. doi: 10.1016/s1053-2498(01)00360-6 11834356

[12] Burton CM Iversen M Carlsen J Mortensen J Andersen CB Steinbruchel D. Acute cellular rejection is a risk factor for bronchiolitis obliterans syndrome independent of post-transplant baseline Fev1. J Heart Lung Transplant (2009) 28(9):888–93. doi: 10.1016/j.healun.2009.04.022 19716040

[13] Verleden SE Ruttens D Vandermeulen E Vaneylen A Dupont LJ Van Raemdonck DE. Bronchiolitis obliterans syndrome and restrictive allograft syndrome: Do risk factors differ? Transplantation (2013) 95(9):1167–72. doi: 10.1097/TP.0b013e318286e076 23425818

[14] Sato M Waddell TK Wagnetz U Roberts HC Hwang DM Haroon A. Restrictive allograft syndrome (Ras): A novel form of chronic lung allograft dysfunction. J Heart Lung Transplant (2011) 30(7):735–42. doi: 10.1016/j.healun.2011.01.712 21419659

[15] Fuehner T Simon A Dierich M Dewall C Laenger F Pletz MW. Indicators for steroid response in biopsy proven acute graft rejection after lung transplantation. Respir Med (2009) 103(8):1114–21. doi: 10.1016/j.rmed.2009.03.013 19398195

[16] Witt CA Gaut JP Yusen RD Byers DE Iuppa JA Bennett Bain K. Acute antibody-mediated rejection after lung transplantation. J Heart Lung Transplant (2013) 32(10):1034–40. doi: 10.1016/j.healun.2013.07.004 PMC3822761 23953920

[17] Roux A Bendib Le Lan I Holifanjaniaina S Thomas KA Hamid AM Picard C. Antibody-mediated rejection in lung transplantation: Clinical outcomes and donor-specific antibody characteristics. Am J Transplant (2016) 16(4):1216–28. doi: 10.1111/ajt.13589 26845386

[18] Khush KK Potena L Cherikh WS Chambers DC Harhay MO Hayes D Jr.. The international thoracic organ transplant registry of the international society for heart and lung transplantation: 37th adult heart transplantation report-2020; focus on deceased donor characteristics. J Heart Lung Transplant (2020) 39(10):1003–15. doi: 10.1016/j.healun.2020.07.010 PMC7737223 32811772

[19] Tanaka S Gauthier JM Terada Y Takahashi T Li W Hashimoto K. Bacterial products in donor airways prevent the induction of lung transplant tolerance. Am J Transplant (2021) 21(1):353–61. doi: 10.1111/ajt.16256 PMC7775268 32786174

[20] Bazemore K Rohly M Permpalung N Yu K Timofte I Brown AW. Donor derived cell free DNA% is elevated with pathogens that are risk factors for acute and chronic lung allograft injury. J Heart Lung Transplant (2021) 40(11):1454–62. doi: 10.1016/j.healun.2021.05.012 PMC8571060 34344623

[21] Gunasekaran M Bansal S Ravichandran R Sharma M Perincheri S Rodriguez F. Respiratory viral infection in lung transplantation induces exosomes that trigger chronic rejection. J Heart Lung Transplant (2020) 39(4):379–88. doi: 10.1016/j.healun.2019.12.009 PMC7102671 32033844

[22] Amubieya O Ramsey A DerHovanessian A Fishbein GA Lynch JP 3rd Belperio JA. Chronic lung allograft dysfunction: Evolving concepts and therapies. Semin Respir Crit Care Med (2021) 42(3):392–410. doi: 10.1055/s-0041-1729175 34030202

[23] Verleden GM Glanville AR Lease ED Fisher AJ Calabrese F Corris PA. Chronic lung allograft dysfunction: Definition, diagnostic criteria, and approaches to treatment-a consensus report from the pulmonary council of the ishlt. J Heart Lung Transplant (2019) 38(5):493–503. doi: 10.1016/j.healun.2019.03.009 30962148

[24] Stewart S Fishbein MC Snell GI Berry GJ Boehler A Burke MM. Revision of the 1996 working formulation for the standardization of nomenclature in the diagnosis of lung rejection. J Heart Lung Transplant (2007) 26(12):1229–42. doi: 10.1016/j.healun.2007.10.017 18096473

[25] Kreisel D Sugimoto S Zhu J Nava R Li W Okazaki M. Emergency granulopoiesis promotes neutrophil-dendritic cell encounters that prevent mouse lung allograft acceptance. Blood (2011) 118(23):6172–82. doi: 10.1182/blood-2011-04-347823 PMC3234670 21972291

[26] Witt CA Puri V Gelman AE Krupnick AS Kreisel D. Lung transplant immunosuppression - time for a new approach? Expert Rev Clin Immunol (2014) 10(11):1419–21. doi: 10.1586/1744666X.2014.959499 PMC4412736 25220652

[27] Porrett PM Yuan X LaRosa DF Walsh PT Yang J Gao W. Mechanisms underlying blockade of allograft acceptance by tlr ligands. J Immunol (2008) 181(3):1692–9. doi: 10.4049/jimmunol.181.3.1692 PMC2840047 18641305

[28] Gelman AE Li W Richardson SB Zinselmeyer BH Lai J Okazaki M. Cutting edge: Acute lung allograft rejection is independent of secondary lymphoid organs. J Immunol (2009) 182(7):3969–73. doi: 10.4049/jimmunol.0803514 PMC3760174 19299693

[29] Krupnick AS Lin X Li W Higashikubo R Zinselmeyer BH Hartzler H. Central memory Cd8+ T lymphocytes mediate lung allograft acceptance. J Clin Invest (2014) 124(3):1130–43. doi: 10.1172/JCI71359 PMC3938255 24569377

[30] Li W Bribriesco AC Nava RG Brescia AA Ibricevic A Spahn JH. Lung transplant acceptance is facilitated by early events in the graft and is associated with lymphoid neogenesis. Mucosal Immunol (2012) 5(5):544–54. doi: 10.1038/mi.2012.30 PMC3425714 22549742

[31] Takahashi T Hsiao HM Tanaka S Li W Higashikubo R Scozzi D. Pd-1 expression on Cd8(+) T cells regulates their differentiation within lung allografts and is critical for tolerance induction. Am J Transplant (2018) 18(1):216–25. doi: 10.1111/ajt.14437 PMC5739961 28730633

[32] Snyder ME Finlayson MO Connors TJ Dogra P Senda T Bush E. Generation and persistence of human tissue-resident memory T cells in lung transplantation. Sci Immunol (2019) 4(33):eaav5581. doi: 10.1126/sciimmunol.aav5581 30850393 PMC6435356

[33] Nicosia M Fairchild RL Valujskikh A. Memory T cells in transplantation: Old challenges define new directions. Transplantation (2020) 104(10):2024–34. doi: 10.1097/TP.0000000000003169 PMC7416526 32039966

[34] Leonard DA Amin KR Giele H Fildes JE Wong JKF. Skin immunology and rejection in vca and organ transplantation. Curr Transplant Rep (2020) 7(4):251–9. doi: 10.1007/s40472-020-00310-1

[35] Lakkis FG Arakelov A Konieczny BT Inoue Y. Immunologic 'Ignorance' of vascularized organ transplants in the absence of secondary lymphoid tissue. Nat Med (2000) 6(6):686–8. doi: 10.1038/76267 10835686

[36] Zhang N Schroppel B Lal G Jakubzick C Mao X Chen D. Regulatory T cells sequentially migrate from inflamed tissues to draining lymph nodes to suppress the alloimmune response. Immunity (2009) 30(3):458–69. doi: 10.1016/j.immuni.2008.12.022 PMC2737741 19303390

[37] Gelman AE Okazaki M Sugimoto S Li W Kornfeld CG Lai J. Ccr2 regulates monocyte recruitment as well as Cd4 T1 allorecognition after lung transplantation. Am J Transplant (2010) 10(5):1189–99. doi: 10.1111/j.1600-6143.2010.03101.x PMC3746750 20420631

[38] Todd JL Wang X Sugimoto S Kennedy VE Zhang HL Pavlisko EN. Hyaluronan contributes to bronchiolitis obliterans syndrome and stimulates lung allograft rejection through activation of innate immunity. Am J Respir Crit Care Med (2014) 189(5):556–66. doi: 10.1164/rccm.201308-1481OC PMC3977710 24471427

[39] Kreisel D Nava RG Li W Zinselmeyer BH Wang B Lai J. *In vivo* two-photon imaging reveals monocyte-dependent neutrophil extravasation during pulmonary inflammation. Proc Natl Acad Sci USA (2010) 107(42):18073–8. doi: 10.1073/pnas.1008737107 PMC2964224 20923880

[40] Bharat A Kreisel D. Immunopathogenesis of primary graft dysfunction after lung transplantation. Ann Thorac Surg (2018) 105(3):671–4. doi: 10.1016/j.athoracsur.2017.11.007 29455798

[41] Zheng Z Chiu S Akbarpour M Sun H Reyfman PA Anekalla KR. Donor pulmonary intravascular nonclassical monocytes recruit recipient neutrophils and mediate primary lung allograft dysfunction. Sci Transl Med (2017) 9(394):eaal4508. doi: 10.1126/scitranslmed.aal4508 28615357 PMC5568853

[42] Burlingham WJ Love RB Jankowska-Gan E Haynes LD Xu Q Bobadilla JL. Il-17-Dependent cellular immunity to collagen type V predisposes to obliterative bronchiolitis in human lung transplants. J Clin Invest (2007) 117(11):3498–506. doi: 10.1172/JCI28031 PMC2040314 17965778

[43] Koutsokera A Royer PJ Antonietti JP Fritz A Benden C Aubert JD. Development of a multivariate prediction model for early-onset bronchiolitis obliterans syndrome and restrictive allograft syndrome in lung transplantation. Front Med (Lausanne) (2017) 4:109. doi: 10.3389/fmed.2017.00109 28770204 PMC5511826

[44] Balsara KR Krupnick AS Bell JM Khiabani A Scavuzzo M Hachem R. A single-center experience of 1500 lung transplant patients. J Thorac Cardiovasc Surg (2018) 156(2):894–905.e3. doi: 10.1016/j.jtcvs.2018.03.112 29891245 PMC6939473

[45] Christie JD Kotloff RM Ahya VN Tino G Pochettino A Gaughan C. The effect of primary graft dysfunction on survival after lung transplantation. Am J Respir Crit Care Med (2005) 171(11):1312–6. doi: 10.1164/rccm.200409-1243OC PMC2718464 15764726

[46] Yeung JC Krueger T Yasufuku K de Perrot M Pierre AF Waddell TK. Outcomes after transplantation of lungs preserved for more than 12 h: A retrospective study. Lancet Respir Med (2017) 5(2):119–24. doi: 10.1016/S2213-2600(16)30323-X 27866861

[47] den Hengst WA Gielis JF Lin JY Van Schil PE De Windt LJ Moens AL. Lung ischemia-reperfusion injury: A molecular and clinical view on a complex pathophysiological process. Am J Physiol Heart Circ Physiol (2010) 299(5):H1283–99. doi: 10.1152/ajpheart.00251.2010 20833966

[48] Fisher AB Dodia C Tan ZT Ayene I Eckenhoff RG. Oxygen-dependent lipid peroxidation during lung ischemia. J Clin Invest (1991) 88(2):674–9. doi: 10.1172/JCI115352 PMC295411 1864976

[49] Krishnadasan B Naidu BV Byrne K Fraga C Verrier ED Mulligan MS. The role of proinflammatory cytokines in lung ischemia-reperfusion injury. J Thorac Cardiovasc Surg (2003) 125(2):261–72. doi: 10.1067/mtc.2003.16 12579094

[50] Yano M Omoto Y Yamakawa Y Nakashima Y Kiriyama M Saito Y. Increased matrix metalloproteinase 9 activity and mrna expression in lung ischemia-reperfusion injury. J Heart Lung Transplant (2001) 20(6):679–86. doi: 10.1016/s1053-2498(01)00250-9 11404174

[51] Naidu BV Krishnadasan B Farivar AS Woolley SM Thomas R Van Rooijen N. Early activation of the alveolar macrophage is critical to the development of lung ischemia-reperfusion injury. J Thorac Cardiovasc Surg (2003) 126(1):200–7. doi: 10.1016/s0022-5223(03)00390-8 12878956

[52] Land WG Agostinis P Gasser S Garg AD Linkermann A. Transplantation and damage-associated molecular patterns (Damps). Am J Transplant (2016) 16(12):3338–61. doi: 10.1111/ajt.13963 27421829

[53] Laubach VE Sharma AK. Mechanisms of lung ischemia-reperfusion injury. Curr Opin Organ Transplant (2016) 21(3):246–52. doi: 10.1097/MOT.0000000000000304 PMC4861054 26945320

[54] Uehara M Solhjou Z Banouni N Kasinath V Xiaqun Y Dai L. Ischemia augments alloimmune injury through il-6-Driven Cd4(+) alloreactivity. Sci Rep (2018) 8(1):2461. doi: 10.1038/s41598-018-20858-4 29410442 PMC5802749

[55] Cypel M Rubacha M Yeung J Hirayama S Torbicki K Madonik M. Normothermic *ex vivo* perfusion prevents lung injury compared to extended cold preservation for transplantation. Am J Transplant (2009) 9(10):2262–9. doi: 10.1111/j.1600-6143.2009.02775.x 19663886

[56] Steen S Sjoberg T Pierre L Liao Q Eriksson L Algotsson L. Transplantation of lungs from a non-Heart-Beating donor. Lancet (2001) 357(9259):825–9. doi: 10.1016/S0140-6736(00)04195-7 11265950

[57] Niikawa H Okamoto T Ayyat KS Itoda Y Farver CF McCurry KR. The protective effect of prone lung position on ischemia-reperfusion injury and lung function in an *ex vivo* porcine lung model. J Thorac Cardiovasc Surg (2019) 157(1):425–33. doi: 10.1016/j.jtcvs.2018.08.101 30415898

[58] Buchko MT Boroumand N Cheng JC Hirji A Halloran K Freed DH. Clinical transplantation using negative pressure ventilation *ex situ* lung perfusion with extended criteria donor lungs. Nat Commun (2020) 11(1):5765. doi: 10.1038/s41467-020-19581-4 33188221 PMC7666579

[59] Arni S Maeyashiki T Citak N Opitz I Inci I. Subnormothermic *ex vivo* lung perfusion temperature improves graft preservation in lung transplantation. Cells (2021) 10(4):748. doi: 10.3390/cells10040748 33805274 PMC8067331

[60] Gloria JN Yerxa J Kesseli SJ Davis RP Samoylova ML Barbas AS. Subnormothermic *ex vivo* lung perfusion attenuates graft inflammation in a rat transplant model. J Thorac Cardiovasc Surg (2021) S0022-5223(21)00165-3. doi: 10.1016/j.jtcvs.2021.01.066 33640121

[61] Iske J Hinze CA Salman J Haverich A Tullius SG Ius F. The potential of *ex vivo* lung perfusion on improving organ quality and ameliorating ischemia reperfusion injury. Am J Transplant (2021) 21(12):3831–9. doi: 10.1111/ajt.16784 PMC8925042 34355495

[62] Mazzeo AT Fanelli V Boffini M Medugno M Filippini C Simonato E. Feasibility of lung microdialysis to assess metabolism during clinical *ex vivo* lung perfusion. J Heart Lung Transplant (2019) 38(3):267–76. doi: 10.1016/j.healun.2018.12.015 30642797

[63] Yeung JC Cypel M Machuca TN Koike T Cook DJ Bonato R. Physiologic assessment of the *ex vivo* donor lung for transplantation. J Heart Lung Transplant (2012) 31(10):1120–6. doi: 10.1016/j.healun.2012.08.016 22975103

[64] Takahashi M Andrew Cheung HY Watanabe T Zamel R Cypel M Liu M. Strategies to prolong homeostasis of *ex vivo* perfused lungs. J Thorac Cardiovasc Surg (2021) 161(6):1963–73. doi: 10.1016/j.jtcvs.2020.07.104 32958268

[65] Prasad NK Pasrija C Talaie T Krupnick AS Zhao Y Lau CL. *Ex vivo* lung perfusion: Current achievements and future directions. Transplantation (2021) 105(5):979–85. doi: 10.1097/TP.0000000000003483 PMC8792510 33044428

[66] Gilmour J Griffiths C Pither T Scott WE 3rd Fisher AJ. Normothermic machine perfusion of donor-lungs *ex-vivo*: Promoting clinical adoption. Curr Opin Organ Transplant (2020) 25(3):285–92. doi: 10.1097/MOT.0000000000000765 32304426

[67] Stone JP Ball AL Crichley W Yonan N Liao Q Sjoberg T. *ex vivo* lung perfusion improves the inflammatory signaling profile of the porcine donor lung following transplantation. Transplantation (2020) 104(9):1899–905. doi: 10.1097/TP.0000000000003338 32502131

[68] Stone JP Critchley WR Major T Rajan G Risnes I Scott H. Altered immunogenicity of donor lungs *via* removal of passenger leukocytes using *ex vivo* lung perfusion. Am J Transplant (2016) 16(1):33–43. doi: 10.1111/ajt.13446 26366523

[69] Dromparis P Aboelnazar NS Wagner S Himmat S White CW Hatami S. *Ex vivo* perfusion induces a time- and perfusate-dependent molecular repair response in explanted porcine lungs. Am J Transplant (2019) 19(4):1024–36. doi: 10.1111/ajt.15123 30230229

[70] Mulloy DP Stone ML Crosby IK Lapar DJ Sharma AK Webb DV. *Ex vivo* rehabilitation of non-Heart-Beating donor lungs in preclinical porcine model: Delayed perfusion results in superior lung function. J Thorac Cardiovasc Surg (2012) 144(5):1208–15. doi: 10.1016/j.jtcvs.2012.07.056 PMC3477251 22944084

[71] Lin H Chen M Tian F Tikkanen J Ding L Andrew Cheung HY. Alpha1-Anti-Trypsin improves function of porcine donor lungs during *ex-vivo* lung perfusion. J Heart Lung Transplant (2018) 37(5):656–66. doi: 10.1016/j.healun.2017.09.019 29153638

[72] Stone ML Sharma AK Mas VR Gehrau RC Mulloy DP Zhao Y. *Ex vivo* perfusion with adenosine A2a receptor agonist enhances rehabilitation of murine donor lungs after circulatory death. Transplantation (2015) 99(12):2494–503. doi: 10.1097/TP.0000000000000830 PMC4668207 26262504

[73] Emaminia A Lapar DJ Zhao Y Steidle JF Harris DA Laubach VE. Adenosine a(2)a agonist improves lung function during *ex vivo* lung perfusion. Ann Thorac Surg (2011) 92(5):1840–6. doi: 10.1016/j.athoracsur.2011.06.062 PMC3259746 22051279

[74] Huerter ME Sharma AK Zhao Y Charles EJ Kron IL Laubach VE. Attenuation of pulmonary ischemia-reperfusion injury by adenosine A2b receptor antagonism. Ann Thorac Surg (2016) 102(2):385–93. doi: 10.1016/j.athoracsur.2016.02.060 PMC4958568 27109193

[75] Arni S Maeyashiki T Latshang T Opitz I Inci I. *ex vivo* lung perfusion with K(Atp) channel modulators antagonize ischemia reperfusion injury. Cells (2021) 10(9):2296. doi: 10.3390/cells10092296 34571948 PMC8472464

[76] Wang X Wang Y Parapanov R Abdelnour E Gronchi F Perentes JY. Pharmacological reconditioning of marginal donor rat lungs using inhibitors of peroxynitrite and poly (Adp-ribose) polymerase during *ex vivo* lung perfusion. Transplantation (2016) 100(7):1465–73. doi: 10.1097/TP.0000000000001183 27331361

[77] Yamada Y Iskender I Arni S Hillinger S Cosgun T Yu K. *Ex vivo* treatment with inhaled n-acetylcysteine in porcine lung transplantation. J Surg Res (2017) 218:341–7. doi: 10.1016/j.jss.2017.06.061 28985871

[78] Magruder JT Grimm JC Crawford TC Johnston L Santhanam L Stephens RS. Imatinib is protective against ischemia-reperfusion injury in an *ex vivo* rabbit model of lung injury. Ann Thorac Surg (2018) 105(3):950–6. doi: 10.1016/j.athoracsur.2017.10.002 29289364

[79] Wang X Parapanov R Debonneville A Wang Y Abdelnour-Berchtold E Gonzalez M. Treatment with 3-aminobenzamide during *ex vivo* lung perfusion of damaged rat lungs reduces graft injury and dysfunction after transplantation. Am J Transplant (2020) 20(4):967–76. doi: 10.1111/ajt.15695 31710417

[80] Martens A Boada M Vanaudenaerde BM Verleden SE Vos R Verleden GM. Steroids can reduce warm ischemic reperfusion injury in a porcine donation after circulatory death model with *ex vivo* lung perfusion evaluation. Transpl Int (2016) 29(11):1237–46. doi: 10.1111/tri.12823 27514498

[81] Machuca TN Cypel M Bonato R Yeung JC Chun YM Juvet S. Safety and efficacy of *ex vivo* donor lung adenoviral il-10 gene therapy in a Large animal lung transplant survival model. Hum Gene Ther (2017) 28(9):757–65. doi: 10.1089/hum.2016.070 28052693

[82] Francioli C Wang X Parapanov R Abdelnour E Lugrin J Gronchi F. Pyrrolidine dithiocarbamate administered during *ex-vivo* lung perfusion promotes rehabilitation of injured donor rat lungs obtained after prolonged warm ischemia. PloS One (2017) 12(3):e0173916. doi: 10.1371/journal.pone.0173916 28323904 PMC5360331

[83] Harada M Oto T Otani S Miyoshi K Okada M Iga N. A neutrophil elastase inhibitor improves lung function during *ex vivo* lung perfusion. Gen Thorac Cardiovasc Surg (2015) 63(12):645–51. doi: 10.1007/s11748-015-0585-0 26346003

[84] Wang A Ribeiro RVP Ali A Brambate E Abdelnour-Berchtold E Michaelsen V. *Ex vivo* enzymatic treatment converts blood type a donor lungs into universal blood type lungs. Sci Transl Med (2022) 14(632):eabm7190. doi: 10.1126/scitranslmed.abm7190 35171649

[85] Michaelsen VS Ribeiro RVP Ali A Wang A Gazzalle A Keshavjee S. Safety of continuous 12-hour delivery of antimicrobial doses of inhaled nitric oxide during *ex vivo* lung perfusion. J Thorac Cardiovasc Surg (2022) 163(3):841–9.e1. doi: 10.1016/j.jtcvs.2020.11.150 33478833

[86] Nakajima D Watanabe Y Ohsumi A Pipkin M Chen M Mordant P. Mesenchymal stromal cell therapy during *ex vivo* lung perfusion ameliorates ischemia-reperfusion injury in lung transplantation. J Heart Lung Transplant (2019) 38(11):1214–23. doi: 10.1016/j.healun.2019.07.006 31474491

[87] Stone ML Zhao Y Robert Smith J Weiss ML Kron IL Laubach VE. Mesenchymal stromal cell-derived extracellular vesicles attenuate lung ischemia-reperfusion injury and enhance reconditioning of donor lungs after circulatory death. Respir Res (2017) 18(1):212. doi: 10.1186/s12931-017-0704-9 29268735 PMC5740880

[88] Martens A Ordies S Vanaudenaerde BM Verleden SE Vos R Van Raemdonck DE. Immunoregulatory effects of multipotent adult progenitor cells in a porcine *ex vivo* lung perfusion model. Stem Cell Res Ther (2017) 8(1):159. doi: 10.1186/s13287-017-0603-5 28676074 PMC5497348

[89] Mordant P Nakajima D Kalaf R Iskender I Maahs L Behrens P. Mesenchymal stem cell treatment is associated with decreased perfusate concentration of interleukin-8 during *ex vivo* perfusion of donor lungs after 18-hour preservation. J Heart Lung Transplant (2016) 35(10):1245–54. doi: 10.1016/j.healun.2016.04.017 27444694

[90] Lonati C Bassani GA Brambilla D Leonardi P Carlin A Maggioni M. Mesenchymal stem cell-derived extracellular vesicles improve the molecular phenotype of isolated rat lungs during Ischemia/Reperfusion injury. J Heart Lung Transplant (2019) 38(12):1306–16. doi: 10.1016/j.healun.2019.08.016 31530458

[91] Miyamoto E Takahagi A Ohsumi A Martinu T Hwang D Boonstra KM. *Ex vivo* delivery of regulatory T cells for control of alloimmune priming in the donor lung. Eur Respir J (2021) 59(4):2100798. doi: 10.1183/13993003.00798-2021 34475226

[92] Warnecke G Van Raemdonck D Smith MA Massard G Kukreja J Rea F. Normothermic *ex-vivo* preservation with the portable organ care system lung device for bilateral lung transplantation (Inspire): A randomised, open-label, non-inferiority, phase 3 study. Lancet Respir Med (2018) 6(5):357–67. doi: 10.1016/S2213-2600(18)30136-X 29650408

[93] Slama A Schillab L Barta M Benedek A Mitterbauer A Hoetzenecker K. Standard donor lung procurement with normothermic *ex vivo* lung perfusion: A prospective randomized clinical trial. J Heart Lung Transplant (2017) 36(7):744–53. doi: 10.1016/j.healun.2017.02.011 28314503

[94] Loor G Warnecke G Villavicencio MA Smith MA Kukreja J Ardehali A. Portable normothermic *ex-vivo* lung perfusion, ventilation, and functional assessment with the organ care system on donor lung use for transplantation from extended-criteria donors (Expand): A single-arm, pivotal trial. Lancet Respir Med (2019) 7(11):975–84. doi: 10.1016/S2213-2600(19)30200-0 31378427

[95] Cypel M Yeung JC Liu M Anraku M Chen F Karolak W. Normothermic *ex vivo* lung perfusion in clinical lung transplantation. N Engl J Med (2011) 364(15):1431–40. doi: 10.1056/NEJMoa1014597 21488765

[96] Fisher A Andreasson A Chrysos A Lally J Mamasoula C Exley C. An observational study of donor *ex vivo* lung perfusion in uk lung transplantation: Develop-uk. Health Technol Assess (2016) 20(85):1–276. doi: 10.3310/hta20850 PMC5136735 27897967

[97] Nilsson T Wallinder A Henriksen I Nilsson JC Ricksten SE Moller-Sorensen H. Lung transplantation after *ex vivo* lung perfusion in two Scandinavian centres. Eur J Cardiothorac Surg (2019) 55(4):766–72. doi: 10.1093/ejcts/ezy354 PMC6421510 30376058

[98] Sanchez PG Cantu E Hartwig M D’Ovidio F Machuca T Whitson B. The novel study. A multi-center clinical trial studying the safety of *ex vivo* lung perfusion. J Heart Lung Transplant (2020) 39(4):S110. doi: 10.1016/j.healun.2020.01.977

[99] Guenthart BA O'Neill JD Kim J Queen D Chicotka S Fung K. Regeneration of severely damaged lungs using an interventional cross-circulation platform. Nat Commun (2019) 10(1):1985. doi: 10.1038/s41467-019-09908-1 31064987 PMC6504972

[100] O’Neill JD Guenthart BA Kim J Chicotka S Queen D Fung K. Cross-circulation for extracorporeal support and recovery of the lung. Nat Biomed Eng (2017) 1(3):37. doi: 10.1038/s41551-017-0037

[101] Hozain AE Tipograf Y Pinezich MR Cunningham KM Donocoff R Queen D. Multiday maintenance of extracorporeal lungs using cross-circulation with conscious swine. J Thorac Cardiovasc Surg (2020) 159(4):1640–53.e18. doi: 10.1016/j.jtcvs.2019.09.121 31761338 PMC7094131

[102] Hozain AE O'Neill JD Pinezich MR Tipograf Y Donocoff R Cunningham KM. Xenogeneic cross-circulation for extracorporeal recovery of injured human lungs. Nat Med (2020) 26(7):1102–13. doi: 10.1038/s41591-020-0971-8 PMC9990469 32661401

[103] O'Neill JD Guenthart BA Hozain AE Bacchetta M. Xenogeneic support for the recovery of human donor organs. J Thorac Cardiovasc Surg (2021) 163(4):1563–70. doi: 10.1016/j.jtcvs.2021.07.055 34607726

[104] Ali A Wang A Ribeiro RVP Beroncal EL Baciu C Galasso M. Static lung storage at 10 degrees c maintains mitochondrial health and preserves donor organ function. Sci Transl Med (2021) 13(611):eabf7601. doi: 10.1126/scitranslmed.abf7601 34524862

[105] Kayawake H Chen-Yoshikawa TF Saito M Yamagishi H Yoshizawa A Hirano SI. Protective effects of a hydrogen-rich preservation solution in a canine lung transplantation model. Ann Thorac Surg (2021) 111(1):246–52. doi: 10.1016/j.athoracsur.2020.05.076 32649946

[106] Saito M Chen-Yoshikawa TF Takahashi M Kayawake H Yokoyama Y Kurokawa R. Protective effects of a hydrogen-rich solution during cold ischemia in rat lung transplantation. J Thorac Cardiovasc Surg (2020) 159(5):2110–8. doi: 10.1016/j.jtcvs.2019.09.175 31780065

[107] Friedenstein AJ Chailakhjan RK Lalykina KS. The development of fibroblast colonies in monolayer cultures of Guinea-pig bone marrow and spleen cells. Cell Tissue Kinet (1970) 3(4):393–403. doi: 10.1111/j.1365-2184.1970.tb00347.x 5523063

[108] Dominici M Le Blanc K Mueller I Slaper-Cortenbach I Marini F Krause D. Minimal criteria for defining multipotent mesenchymal stromal cells. the international society for cellular therapy position statement. Cytotherapy (2006) 8(4):315–7. doi: 10.1080/14653240600855905 16923606

[109] Sabatini F Petecchia L Tavian M Jodon de Villeroche V Rossi GA Brouty-Boye D. Human bronchial fibroblasts exhibit a mesenchymal stem cell phenotype and multilineage differentiating potentialities. Lab Invest (2005) 85(8):962–71. doi: 10.1038/labinvest.3700300 15924148

[110] Mendez-Ferrer S Michurina TV Ferraro F Mazloom AR Macarthur BD Lira SA. Mesenchymal and haematopoietic stem cells form a unique bone marrow niche. Nature (2010) 466(7308):829–34. doi: 10.1038/nature09262 PMC3146551 20703299

[111] Tropea KA Leder E Aslam M Lau AN Raiser DM Lee JH. Bronchioalveolar stem cells increase after mesenchymal stromal cell treatment in a mouse model of bronchopulmonary dysplasia. Am J Physiol Lung Cell Mol Physiol (2012) 302(9):L829–37. doi: 10.1152/ajplung.00347.2011 PMC3362163 22328358

[112] Lama VN Smith L Badri L Flint A Andrei AC Murray S. Evidence for tissue-resident mesenchymal stem cells in human adult lung from studies of transplanted allografts. J Clin Invest (2007) 117(4):989–96. doi: 10.1172/JCI29713 PMC1810571 17347686

[113] Li J Ezzelarab MB Cooper DK. Do mesenchymal stem cells function across species barriers? Relevance for xenotransplantation. Xenotransplantation (2012) 19(5):273–85. doi: 10.1111/xen.12000 PMC3445044 22978461

[114] Naserian S Shamdani S Arouche N Uzan G. Regulatory T cell induction by mesenchymal stem cells depends on the expression of Tnfr2 by T cells. Stem Cell Res Ther (2020) 11(1):534. doi: 10.1186/s13287-020-02057-z 33303019 PMC7731479

[115] Duffy MM Ritter T Ceredig R Griffin MD. Mesenchymal stem cell effects on T-cell effector pathways. Stem Cell Res Ther (2011) 2(4):34. doi: 10.1186/scrt75 21861858 PMC3219065

[116] Aggarwal S Pittenger MF. Human mesenchymal stem cells modulate allogeneic immune cell responses. Blood (2005) 105(4):1815–22. doi: 10.1182/blood-2004-04-1559 15494428

[117] Beldi G Bahiraii S Lezin C Nouri Barkestani M Abdelgawad ME Uzan G. Tnfr2 is a crucial hub controlling mesenchymal stem cell biological and functional properties. Front Cell Dev Biol (2020) 8:596831. doi: 10.3389/fcell.2020.596831 33344453 PMC7746825

[118] Wu X Jiang J Gu Z Zhang J Chen Y Liu X. Mesenchymal stromal cell therapies: Immunomodulatory properties and clinical progress. Stem Cell Res Ther (2020) 11(1):345. doi: 10.1186/s13287-020-01855-9 32771052 PMC7414268

[119] Bulati M Miceli V Gallo A Amico G Carcione C Pampalone M. The immunomodulatory properties of the human amnion-derived mesenchymal Stromal/Stem cells are induced by inf-gamma produced by activated lymphomonocytes and are mediated by cell-to-Cell contact and soluble factors. Front Immunol (2020) 11:54. doi: 10.3389/fimmu.2020.00054 32117234 PMC7028706

[120] Poggi A Zocchi MR. Immunomodulatory properties of mesenchymal stromal cells: Still unresolved "Yin and yang". Curr Stem Cell Res Ther (2019) 14(4):344–50. doi: 10.2174/1574888X14666181205115452 30516112

[121] Hwang B Liles WC Waworuntu R Mulligan MS. Pretreatment with bone marrow-derived mesenchymal stromal cell-conditioned media confers pulmonary ischemic tolerance. J Thorac Cardiovasc Surg (2016) 151(3):841–9. doi: 10.1016/j.jtcvs.2015.11.043 PMC4761408 26896360

[122] Stavely R Nurgali K. The emerging antioxidant paradigm of mesenchymal stem cell therapy. Stem Cells Transl Med (2020) 9(9):985–1006. doi: 10.1002/sctm.19-0446 32497410 PMC7445024

[123] Miceli V Bertani A. Mesenchymal Stromal/Stem cells and their products as a therapeutic tool to advance lung transplantation. Cells (2022) 11(5):826. doi: 10.3390/cells11050826 35269448 PMC8909054

[124] Sinclair K Yerkovich ST Chambers DC. Mesenchymal stem cells and the lung. Respirology (2013) 18(3):397–411. doi: 10.1111/resp.12050 23316733

[125] Eggenhofer E Benseler V Kroemer A Popp FC Geissler EK Schlitt HJ. Mesenchymal stem cells are short-lived and do not migrate beyond the lungs after intravenous infusion. Front Immunol (2012) 3:297. doi: 10.3389/fimmu.2012.00297 23056000 PMC3458305

[126] Matthay MA Calfee CS Zhuo H Thompson BT Wilson JG Levitt JE. Treatment with allogeneic mesenchymal stromal cells for moderate to severe acute respiratory distress syndrome (Start study): A randomised phase 2a safety trial. Lancet Respir Med (2019) 7(2):154–62. doi: 10.1016/s2213-2600(18)30418-1 PMC7597675 30455077

[127] Lalu MM McIntyre L Pugliese C Fergusson D Winston BW Marshall JC. Safety of cell therapy with mesenchymal stromal cells (Safecell): A systematic review and meta-analysis of clinical trials. PloS One (2012) 7(10):e47559. doi: 10.1371/journal.pone.0047559 23133515 PMC3485008

[128] Keller CA Gonwa TA Hodge DO Hei DJ Centanni JM Zubair AC. Feasibility, safety, and tolerance of mesenchymal stem cell therapy for obstructive chronic lung allograft dysfunction. Stem Cells Transl Med (2018) 7(2):161–7. doi: 10.1002/sctm.17-0198 PMC5788872 29322685

[129] Chambers DC Enever D Lawrence S Sturm MJ Herrmann R Yerkovich S. Mesenchymal stromal cell therapy for chronic lung allograft dysfunction: Results of a first-in-Man study. Stem Cells Transl Med (2017) 6(4):1152–7. doi: 10.1002/sctm.16-0372 PMC5442848 28186707

[130] Thery C Witwer KW Aikawa E Alcaraz MJ Anderson JD Andriantsitohaina R. Minimal information for studies of extracellular vesicles 2018 (Misev2018): A position statement of the international society for extracellular vesicles and update of the Misev2014 guidelines. J Extracell Vesicles (2018) 7(1):1535750. doi: 10.1080/20013078.2018.1535750 30637094 PMC6322352

[131] Hayes M Curley GF Masterson C Devaney J O'Toole D Laffey JG. Mesenchymal stromal cells are more effective than the msc secretome in diminishing injury and enhancing recovery following ventilator-induced lung injury. Intensive Care Med Exp (2015) 3(1):29. doi: 10.1186/s40635-015-0065-y 26472334 PMC4607685

[132] Guillamat-Prats R Camprubi-Rimblas M Puig F Herrero R Tantinya N Serrano-Mollar A. Alveolar type ii cells or mesenchymal stem cells: Comparison of two different cell therapies for the treatment of acute lung injury in rats. Cells (2020) 9(8):1816. doi: 10.3390/cells9081816 PMC7464506 32751857

[133] Ionescu L Byrne RN van Haaften T Vadivel A Alphonse RS Rey-Parra GJ. Stem cell conditioned medium improves acute lung injury in mice: *In vivo* evidence for stem cell paracrine action. Am J Physiol Lung Cell Mol Physiol (2012) 303(11):L967–77. doi: 10.1152/ajplung.00144.2011 PMC3532523 23023971

[134] Pacienza N Santa-Cruz D Malvicini R Robledo O Lemus-Larralde G Bertolotti A. Mesenchymal stem cell therapy facilitates donor lung preservation by reducing oxidative damage during ischemia. Stem Cells Int (2019) 2019:8089215. doi: 10.1155/2019/8089215 31481974 PMC6701419

[135] Zhu YG Feng XM Abbott J Fang XH Hao Q Monsel A. Human mesenchymal stem cell microvesicles for treatment of escherichia coli endotoxin-induced acute lung injury in mice. Stem Cells (2014) 32(1):116–25. doi: 10.1002/stem.1504 PMC3947321 23939814

[136] Wakayama H Hashimoto N Matsushita Y Matsubara K Yamamoto N Hasegawa Y. Factors secreted from dental pulp stem cells show multifaceted benefits for treating acute lung injury in mice. Cytotherapy (2015) 17(8):1119–29. doi: 10.1016/j.jcyt.2015.04.009 26031744

[137] Shologu N Scully M Laffey JG O'Toole D. Human mesenchymal stem cell secretome from bone marrow or adipose-derived tissue sources for treatment of hypoxia-induced pulmonary epithelial injury. Int J Mol Sci (2018) 19(10):2996. doi: 10.3390/ijms19102996 PMC6212866 30274394

[138] Tseng N Lambie SC Huynh CQ Sanford B Patel M Herson PS. Mitochondrial transfer from mesenchymal stem cells improves neuronal metabolism after oxidant injury *in vitro*: The role of Miro1. J Cereb Blood Flow Metab (2021) 41(4):761–70. doi: 10.1177/0271678X20928147 PMC7983509 32501156

[139] Lin KC Yeh JN Chen YL Chiang JY Sung PH Lee FY. Xenogeneic and allogeneic mesenchymal stem cells effectively protect the lung against ischemia-reperfusion injury through downregulating the inflammatory, oxidative stress, and autophagic signaling pathways in rat. Cell Transplant (2020) 29:963689720954140. doi: 10.1177/0963689720954140 33050736 PMC7784512

[140] Kim YS Kim JY Cho R Shin DM Lee SW Oh YM. Adipose stem cell-derived nanovesicles inhibit emphysema primarily *via* an Fgf2-dependent pathway. Exp Mol Med (2017) 49(1):e284. doi: 10.1038/emm.2016.127 28082743 PMC5291836

[141] Niroomand A Hirdman G Olm F Lindstedt S. Current status and future perspectives on machine perfusion: A treatment platform to restore and regenerate injured lungs using cell and cytokine adsorption therapy. Cells (2021) 11(1):91. doi: 10.3390/cells11010091 35011653 PMC8750486

[142] Li J Peng Q Yang R Li K Zhu P Zhu Y. Application of mesenchymal stem cells during machine perfusion: An emerging novel strategy for organ preservation. Front Immunol (2021) 12:713920. doi: 10.3389/fimmu.2021.713920 35024039 PMC8744145

[143] Lee JW Fang X Gupta N Serikov V Matthay MA. Allogeneic human mesenchymal stem cells for treatment of *e. coli* endotoxin-induced acute lung injury in the *ex vivo* perfused human lung. Proc Natl Acad Sci USA (2009) 106(38):16357–62. doi: 10.1073/pnas.0907996106 PMC2735560 19721001

[144] Van Raemdonck D Neyrinck A Rega F Devos T Pirenne J. Machine perfusion in organ transplantation: A tool for *ex-vivo* graft conditioning with mesenchymal stem cells? Curr Opin Organ Transplant (2013) 18(1):24–33. doi: 10.1097/MOT.0b013e32835c494f 23254699

[145] Lee JW Krasnodembskaya A McKenna DH Song Y Abbott J Matthay MA. Therapeutic effects of human mesenchymal stem cells in *ex vivo* human lungs injured with live bacteria. Am J Respir Crit Care Med (2013) 187(7):751–60. doi: 10.1164/rccm.201206-0990OC PMC3678109 23292883

[146] McAuley DF Curley GF Hamid UI Laffey JG Abbott J McKenna DH. Clinical grade allogeneic human mesenchymal stem cells restore alveolar fluid clearance in human lungs rejected for transplantation. Am J Physiol Lung Cell Mol Physiol (2014) 306(9):L809–15. doi: 10.1152/ajplung.00358.2013 PMC4010648 24532289

[147] La Francesca S Ting AE Sakamoto J Rhudy J Bonenfant NR Borg ZD. Multipotent adult progenitor cells decrease cold ischemic injury in *ex vivo* perfused human lungs: An initial pilot and feasibility study. Transplant Res (2014) 3(1):19. doi: 10.1186/2047-1440-3-19 25671090 PMC4323223

[148] Gennai S Monsel A Hao Q Park J Matthay MA Lee JW. Microvesicles derived from human mesenchymal stem cells restore alveolar fluid clearance in human lungs rejected for transplantation. Am J Transplant (2015) 15(9):2404–12. doi: 10.1111/ajt.13271 PMC4792255 25847030

[149] Park J Kim S Lim H Liu A Hu S Lee J. Therapeutic effects of human mesenchymal stem cell microvesicles in an *ex vivo* perfused human lung injured with severe. E. Coli Pneumonia Thorax (2019) 74(1):43–50. doi: 10.1136/thoraxjnl-2018-211576 30076187 PMC6295323

[150] Nykanen AI Mariscal A Duong A Estrada C Ali A Hough O. Engineered mesenchymal stromal cell therapy during human lung *ex vivo* lung perfusion is compromised by acidic lung microenvironment. Mol Ther Methods Clin Dev (2021) 23:184–97. doi: 10.1016/j.omtm.2021.05.018 PMC8516994 34703841

[151] Preissler G Loehe F Huff IV Ebersberger U Shuvaev VV Bittmann I. Targeted endothelial delivery of nanosized catalase immunoconjugates protects lung grafts donated after cardiac death. Transplantation (2011) 92(4):380–7. doi: 10.1097/TP.0b013e318226bc6b PMC5505068 21778930

[152] Pickford MA Green CJ Sarathchandra P Fryer PR. Ultrastructural changes in rat lungs after 48 h cold storage with and without reperfusion. Int J Exp Pathol (1990) 71(4):513–28. doi: 10.3389/fphys.2020.581420 PMC2002293 2119217

[153] Jungraithmayr W. Novel strategies for endothelial preservation in lung transplant ischemia-reperfusion injury. Front Physiol (2020) 11:581420. doi: 10.3389/fphys.2020.581420 33391010 PMC7775419

[154] Fiser SM Tribble CG Long SM Kaza AK Cope JT Laubach VE. Lung transplant reperfusion injury involves pulmonary macrophages and circulating leukocytes in a biphasic response. J Thorac Cardiovasc Surg (2001) 121(6):1069–75. doi: 10.1067/mtc.2001.113603 11385373

[155] Fiser SM Tribble CG Long SM Kaza AK Kern JA Kron IL. Pulmonary macrophages are involved in reperfusion injury after lung transplantation. Ann Thorac Surg (2001) 71(4):1134–8. doi: 10.1016/s0003-4975(01)02407-9 11308149

[156] Hidalgo MA Shah KA Fuller BJ Green CJ. Cold ischemia-induced damage to vascular endothelium results in permeability alterations in transplanted lungs. J Thorac Cardiovasc Surg (1996) 112(4):1027–35. doi: 10.1016/S0022-5223(96)70104-6 8873730

[157] Lam CF Liu YC Hsu JK Yeh PA Su TY Huang CC. Autologous transplantation of endothelial progenitor cells attenuates acute lung injury in rabbits. Anesthesiology (2008) 108(3):392–401. doi: 10.1097/ALN.0b013e318164ca64 18292677

[158] Lam CF Roan JN Lee CH Chang PJ Huang CC Liu YC. Transplantation of endothelial progenitor cells improves pulmonary endothelial function and gas exchange in rabbits with endotoxin-induced acute lung injury. Anesth Analg (2011) 112(3):620–7. doi: 10.1213/ANE.0b013e3182075da4 21233499

[159] Yen YT Roan JN Fang SY Chang SW Tseng YL Lam CF. Autologous endothelial progenitor cells improve allograft survival in porcine lung transplantation with prolonged ischemia. Ann Transl Med (2016) 4(15):277. doi: 10.21037/atm.2016.06.22 27570771 PMC4980385

[160] Gao W Jiang T Liu YH Ding WG Guo CC Cui XG. Endothelial progenitor cells attenuate the lung Ischemia/Reperfusion injury following lung transplantation *via* the endothelial nitric oxide synthase pathway. J Thorac Cardiovasc Surg (2019) 157(2):803–14. doi: 10.1016/j.jtcvs.2018.08.092 30391008

[161] Li W Gauthier JM Tong AY Terada Y Higashikubo R Frye CC. Lymphatic drainage from bronchus-associated lymphoid tissue in tolerant lung allografts promotes peripheral tolerance. J Clin Invest (2020) 130(12):6718–27. doi: 10.1172/JCI136057 PMC7685742 33196461

[162] Li W Gauthier JM Higashikubo R Hsiao HM Tanaka S Vuong L. Bronchus-associated lymphoid tissue-resident Foxp3+ T lymphocytes prevent antibody-mediated lung rejection. J Clin Invest (2019) 129(2):556–68. doi: 10.1172/JCI122083 PMC6355223 30561386

[163] Gregson AL Hoji A Saggar R Ross DJ Kubak BM Jamieson BD. Bronchoalveolar immunologic profile of acute human lung transplant allograft rejection. Transplantation (2008) 85(7):1056–9. doi: 10.1097/TP.0b013e318169bd85 PMC2744369 18408589

[164] Ius F Salman J Knoefel AK Sommer W Nakagiri T Verboom M. Increased frequency of Cd4(+) Cd25(High) Cd127(Low) T cells early after lung transplant is associated with improved graft survival - A retrospective study. Transpl Int (2020) 33(5):503–16. doi: 10.1111/tri.13568 31903646

[165] Salman J Ius F Knoefel AK Sommer W Siemeni T Kuehn C. Association of higher Cd4(+) Cd25(High) Cd127(Low) , Foxp3(+) , and il-2(+) T cell frequencies early after lung transplantation with less chronic lung allograft dysfunction at two years. Am J Transplant (2017) 17(6):1637–48. doi: 10.1111/ajt.14148 27931084

[166] Miyamoto E Takahagi A Ohsumi A Martinu T Hwang D Boonstra KM. *Ex vivo* delivery of regulatory T-cells for control of alloimmune priming in the donor lung. Eur Respir J (2022) 59(4):2100798. doi: 10.1183/13993003.00798-2021 34475226

[167] Kaneda H Waddell TK de Perrot M Bai XH Gutierrez C Arenovich T. Pre-implantation multiple cytokine mrna expression analysis of donor lung grafts predicts survival after lung transplantation in humans. Am J Transplant (2006) 6(3):544–51. doi: 10.1111/j.1600-6143.2005.01204.x 16468964

[168] Sage AT Besant JD Mahmoudian L Poudineh M Bai X Zamel R. Fractal circuit sensors enable rapid quantification of biomarkers for donor lung assessment for transplantation. Sci Adv (2015) 1(7):e1500417. doi: 10.1126/sciadv.1500417 26601233 PMC4643795

[169] Verleden SE Martens A Ordies S Neyrinck AP Van Raemdonck DE Verleden GM. Immediate post-operative broncho-alveolar lavage il-6 and il-8 are associated with early outcomes after lung transplantation. Clin Transplant (2018) 32(4):e13219. doi: 10.1111/ctr.13219 29405435

[170] Jong WM Ten Cate H Linnenbank AC de Boer OJ Reitsma PH de Winter RJ. Reduced acute myocardial ischemia-reperfusion injury in il-6-Deficient mice employing a closed-chest model. Inflammation Res (2016) 65(6):489–99. doi: 10.1007/s00011-016-0931-4 PMC4841857 26935770

[171] Batal I Azzi J Mounayar M Abdoli R Moore R Lee JY. The mechanisms of up-regulation of dendritic cell activity by oxidative stress. J Leukoc Biol (2014) 96(2):283–93. doi: 10.1189/jlb.3A0113-033RR PMC4101089 24676276

[172] Salehi S Reed EF. The divergent roles of macrophages in solid organ transplantation. Curr Opin Organ Transplant (2015) 20(4):446–53. doi: 10.1097/MOT.0000000000000209 PMC4520531 26154913

[173] Jiang X Tian W Sung YK Qian J Nicolls MR. Macrophages in solid organ transplantation. Vasc Cell (2014) 6(1):5. doi: 10.1186/2045-824X-6-5 24612731 PMC3975229

[174] Whitehead BF Stoehr C Wu CJ Patterson G Burchard EG Theodore J. Cytokine gene expression in human lung transplant recipients. Transplantation (1993) 56(4):956–61. doi: 10.1097/00007890-199310000-00034 7692639

[175] Iacono A Dauber J Keenan R Spichty K Cai J Grgurich W. Interleukin 6 and interferon-gamma gene expression in lung transplant recipients with refractory acute cellular rejection: Implications for monitoring and inhibition by treatment with aerosolized cyclosporine. Transplantation (1997) 64(2):263–9. doi: 10.1097/00007890-199707270-00015 9256185

[176] Rizzo M SivaSai KS Smith MA Trulock EP Lynch JP Patterson GA. Increased expression of inflammatory cytokines and adhesion molecules by alveolar macrophages of human lung allograft recipients with acute rejection: Decline with resolution of rejection. J Heart Lung Transplant (2000) 19(9):858–65. doi: 10.1016/s1053-2498(00)00165-0 11008075

[177] Yoshida Y Iwaki Y Pham S Dauber JH Yousem SA Zeevi A. Benefits of posttransplantation monitoring of interleukin 6 in lung transplantation. Ann Thorac Surg (1993) 55(1):89–93. doi: 10.1016/0003-4975(93)90479-2 8417717

[178] Wheeler DS Misumi K Walker NM Vittal R Combs MP Aoki Y. Interleukin 6 trans-signaling is a critical driver of lung allograft fibrosis. Am J Transplant (2020) 21(7):2360–71. doi: 10.1111/ajt.16417 PMC8809084 33249747

[179] Garbers C Heink S Korn T Rose-John S. Interleukin-6: Designing specific therapeutics for a complex cytokine. Nat Rev Drug Discov (2018) 17(6):395–412. doi: 10.1038/nrd.2018.45 29725131

[180] Kang S Tanaka T Narazaki M Kishimoto T. Targeting interleukin-6 signaling in clinic. Immunity (2019) 50(4):1007–23. doi: 10.1016/j.immuni.2019.03.026 30995492

[181] Tonsho M Lee S Aoyama A Boskovic S Nadazdin O Capetta K. Tolerance of lung allografts achieved in nonhuman primates *via* mixed hematopoietic chimerism. Am J Transplant (2015) 15(8):2231–9. doi: 10.1111/ajt.13274 PMC4569127 25904524

[182] Vo AA Huang E Ammerman N Toyoda M Ge S Haas M. Clazakizumab for desensitization in highly sensitized patients awaiting transplantation. Am J Transplant (2021) 104(S3):S104–S5. doi: 10.1111/ajt.16926 34910841

[183] Vo AA Choi J Kim I Louie S Cisneros K Kahwaji J. A phase I/Ii trial of the interleukin-6 receptor-specific humanized monoclonal (Tocilizumab) + intravenous immunoglobulin in difficult to desensitize patients. Transplantation (2015) 99(11):2356–63. doi: 10.1097/TP.0000000000000741 26018350

[184] Choi J Aubert O Vo A Loupy A Haas M Puliyanda D. Assessment of tocilizumab (Anti-Interleukin-6 receptor monoclonal) as a potential treatment for chronic antibody-mediated rejection and transplant glomerulopathy in hla-sensitized renal allograft recipients. Am J Transplant (2017) 17(9):2381–9. doi: 10.1111/ajt.14228 28199785

[185] Lavacca A Presta R Gai C Mella A Gallo E Camussi G. Early effects of first-line treatment with anti-Interleukin-6 receptor antibody tocilizumab for chronic active antibody-mediated rejection in kidney transplantation. Clin Transplant (2020) 34(8):e13908. doi: 10.1111/ctr.13908 32415711

[186] Pottebaum AA Venkatachalam K Liu C Brennan DC Murad H Malone AF. Efficacy and safety of tocilizumab in the treatment of acute active antibody-mediated rejection in kidney transplant recipients. Transplant Direct (2020) 6(4):e543. doi: 10.1097/TXD.0000000000000988 32309629 PMC7145000

[187] Chandran S Leung J Hu C Laszik ZG Tang Q Vincenti FG. Interleukin-6 blockade with tocilizumab increases tregs and reduces T effector cytokines in renal graft inflammation: A randomized controlled trial. Am J Transplant (2020) 21(7):2543–54. doi: 10.1111/ajt.16459 33331082

[188] Berastegui C Gomez-Olles S Sanchez-Vidaurre S Culebras M Monforte V Lopez-Meseguer M. Balf cytokines in different phenotypes of chronic lung allograft dysfunction in lung transplant patients. Clin Transplant (2017) 31(3). doi: 10.1111/ctr.12898 28008659

[189] Shino MY Weigt SS Li N Palchevskiy V Derhovanessian A Saggar R. The prognostic importance of Cxcr3 chemokine during organizing pneumonia on the risk of chronic lung allograft dysfunction after lung transplantation. PloS One (2017) 12(7):e0180281. doi: 10.1371/journal.pone.0180281 28686641 PMC5501470

[190] Vincenti F Tedesco Silva H Busque S O'Connell P Friedewald J Cibrik D. Randomized phase 2b trial of tofacitinib (Cp-690,550) in *De novo* kidney transplant patients: Efficacy, renal function and safety at 1 year. Am J Transplant (2012) 12(9):2446–56. doi: 10.1111/j.1600-6143.2012.04127.x 22682022

[191] Southworth T Plumb J Gupta V Pearson J Ramis I Lehner MD. Anti-inflammatory potential of Pi3kdelta and jak inhibitors in asthma patients. Respir Res (2016) 17(1):124. doi: 10.1186/s12931-016-0436-2 27716212 PMC5051065

[192] Singh D Bogus M Moskalenko V Lord R Moran EJ Crater GD. Phase 2 multiple ascending dose study of the inhaled pan-jak inhibitor nezulcitinib (Td-0903) in severe covid-19. Eur Respir J (2021) 58(4):2100673. doi: 10.1183/13993003.00673-2021 34210790 PMC8859971

[193] Zimmermann P Ziesenitz VC Curtis N Ritz N. The immunomodulatory effects of macrolides-A systematic review of the underlying mechanisms. Front Immunol (2018) 9:302. doi: 10.3389/fimmu.2018.00302 29593707 PMC5859047

[194] Vos R Vanaudenaerde BM Verleden SE De Vleeschauwer SI Willems-Widyastuti A Van Raemdonck DE. A randomised controlled trial of azithromycin to prevent chronic rejection after lung transplantation. Eur Respir J (2011) 37(1):164–72. doi: 10.1183/09031936.00068310 20562124

[195] Ruttens D Verleden SE Vandermeulen E Bellon H Vanaudenaerde BM Somers J. Prophylactic azithromycin therapy after lung transplantation: *Post hoc* analysis of a randomized controlled trial. Am J Transplant (2016) 16(1):254–61. doi: 10.1111/ajt.13417 26372728

[196] Jain R Hachem RR Morrell MR Trulock EP Chakinala MM Yusen RD. Azithromycin is associated with increased survival in lung transplant recipients with bronchiolitis obliterans syndrome. J Heart Lung Transplant (2010) 29(5):531–7. doi: 10.1016/j.healun.2009.12.003 PMC2854291 20133163

[197] Federica M Nadia S Monica M Alessandro C Tiberio O Francesco B. Clinical and immunological evaluation of 12-month azithromycin therapy in chronic lung allograft rejection. Clin Transplant (2011) 25(4):E381–9. doi: 10.1111/j.1399-0012.2011.01435.x 21418327

[198] Corris PA Ryan VA Small T Lordan J Fisher AJ Meachery G. A randomised controlled trial of azithromycin therapy in bronchiolitis obliterans syndrome (Bos) post lung transplantation. Thorax (2015) 70(5):442–50. doi: 10.1136/thoraxjnl-2014-205998 PMC4413845 25714615

[199] Van Herck A Frick AE Schaevers V Vranckx A Verbeken EK Vanaudenaerde BM. Azithromycin and early allograft function after lung transplantation: A randomized, controlled trial. J Heart Lung Transplant (2019) 38(3):252–9. doi: 10.1016/j.healun.2018.12.006 30686699

[200] Andreasson AS Karamanou DM Gillespie CS Ozalp F Butt T Hill P. Profiling inflammation and tissue injury markers in perfusate and bronchoalveolar lavage fluid during human *ex vivo* lung perfusion. Eur J Cardiothorac Surg (2017) 51(3):577–86. doi: 10.1093/ejcts/ezw358 PMC5400024 28082471

[201] Charles EJ Huerter ME Wagner CE Sharma AK Zhao Y Stoler MH. Donation after circulatory death lungs transplantable up to six hours after *ex vivo* lung perfusion. Ann Thorac Surg (2016) 102(6):1845–53. doi: 10.1016/j.athoracsur.2016.06.043 PMC5112144 27614736

[202] Haam S Noda K Philips BJ Harano T Sanchez PG Shigemura N. Cyclosporin a administration during *ex vivo* lung perfusion preserves lung grafts in rat transplant model. Transplantation (2020) 104(9):e252–e9. doi: 10.1097/TP.0000000000003237 32217944

[203] Charles EJ Mehaffey JH Sharma AK Zhao Y Stoler MH Isbell JM. Lungs donated after circulatory death and prolonged warm ischemia are transplanted successfully after enhanced *ex vivo* lung perfusion using adenosine A2b receptor antagonism. J Thorac Cardiovasc Surg (2017) 154(5):1811–20. doi: 10.1016/j.jtcvs.2017.02.072 PMC6743073 28483262

[204] Iskender I Arni S Maeyashiki T Citak N Sauer M Rodriguez JM. Perfusate adsorption during *ex vivo* lung perfusion improves early post-transplant lung function. J Thorac Cardiovasc Surg (2021) 161(2):e109–e21. doi: 10.1016/j.jtcvs.2019.12.128 32201002

[205] Gebhardt T Wakim LM Eidsmo L Reading PC Heath WR Carbone FR. Memory T cells in nonlymphoid tissue that provide enhanced local immunity during infection with herpes simplex virus. Nat Immunol (2009) 10(5):524–30. doi: 10.1038/ni.1718 19305395

[206] Steinbach K Vincenti I Merkler D. Resident-memory T cells in tissue-restricted immune responses: For better or worse? Front Immunol (2018) 9:2827. doi: 10.3389/fimmu.2018.02827 30555489 PMC6284001

[207] Schenkel JM Masopust D. Tissue-resident memory T cells. Immunity (2014) 41(6):886–97. doi: 10.1016/j.immuni.2014.12.007 PMC4276131 25526304

[208] Kumar BV Ma W Miron M Granot T Guyer RS Carpenter DJ. Human tissue-resident memory T cells are defined by core transcriptional and functional signatures in lymphoid and mucosal sites. Cell Rep (2017) 20(12):2921–34. doi: 10.1016/j.celrep.2017.08.078 PMC5646692 28930685

[209] Wu H Liao W Li Q Long H Yin H Zhao M. Pathogenic role of tissue-resident memory T cells in autoimmune diseases. Autoimmun Rev (2018) 17(9):906–11. doi: 10.1016/j.autrev.2018.03.014 30005862

[210] Masopust D Soerens AG. Tissue-resident T cells and other resident leukocytes. Annu Rev Immunol (2019) 37:521–46. doi: 10.1146/annurev-immunol-042617-053214 PMC7175802 30726153

[211] Szabo PA Miron M Farber DL. Location, location, location: Tissue resident memory T cells in mice and humans. Sci Immunol (2019) 4(34):eaas9673. doi: 10.1126/sciimmunol.aas9673 30952804 PMC6778482

[212] Mueller SN Mackay LK. Tissue-resident memory T cells: Local specialists in immune defence. Nat Rev Immunol (2016) 16(2):79–89. doi: 10.1038/nri.2015.3 26688350

[213] Turner DL Gordon CL Farber DL. Tissue-resident T cells, in situ immunity and transplantation. Immunol Rev (2014) 258(1):150–66. doi: 10.1111/imr.12149 24517432

[214] Zheng L Orsida B Whitford H Levvey B Ward C Walters EH. Longitudinal comparisons of lymphocytes and subtypes between airway wall and bronchoalveolar lavage after human lung transplantation. Transplantation (2005) 80(2):185–92. doi: 10.1097/01.tp.0000165091.31541.23 16041262

[215] Samat AAK van der Geest J Vastert SJ van Loosdregt J van Wijk F. Tissue-resident memory T cells in chronic inflammation-local cells with systemic effects? Cells (2021) 10(2):409. doi: 10.3390/cells10020409 33669367 PMC7920248

[216] Fonseca R Beura LK Quarnstrom CF Ghoneim HE Fan Y Zebley CC. Developmental plasticity allows outside-in immune responses by resident memory T cells. Nat Immunol (2020) 21(4):412–21. doi: 10.1038/s41590-020-0607-7 PMC7096285 32066954

[217] Gratz IK Campbell DJ. Resident memory T cells show that it is never too late to change your ways. Nat Immunol (2020) 21(4):359–60. doi: 10.1038/s41590-020-0637-1 32205885

[218] Fu J Sykes M. Emerging concepts of tissue-resident memory T cells in transplantation. Transplantation (2021) 106(6):1132–42. doi: 10.1097/TP.0000000000004000 PMC9127003 34873129

[219] Schenk AD Gorbacheva V Rabant M Fairchild RL Valujskikh A. Effector functions of donor-reactive Cd8 memory T cells are dependent on icos induced during division in cardiac grafts. Am J Transplant (2009) 9(1):64–73. doi: 10.1111/j.1600-6143.2008.02460.x 18976292 PMC3289995

[220] Schenk AD Nozaki T Rabant M Valujskikh A Fairchild RL. Donor-reactive Cd8 memory T cells infiltrate cardiac allografts within 24-h posttransplant in naive recipients. Am J Transplant (2008) 8(8):1652–61. doi: 10.1111/j.1600-6143.2008.02302.x PMC2625311 18557725

[221] Koyama I Nadazdin O Boskovic S Ochiai T Smith RN Sykes M. Depletion of Cd8 memory T cells for induction of tolerance of a previously transplanted kidney allograft. Am J Transplant (2007) 7(5):1055–61. doi: 10.1111/j.1600-6143.2006.01703.x PMC3785402 17286617

[222] Donckier V Craciun L Miqueu P Troisi RI Lucidi V Rogiers X. Expansion of memory-type Cd8+ T cells correlates with the failure of early immunosuppression withdrawal after cadaver liver transplantation using high-dose atg induction and rapamycin. Transplantation (2013) 96(3):306–15. doi: 10.1097/TP.0b013e3182985414 23799424

[223] Ascon M Ascon DB Liu M Cheadle C Sarkar C Racusen L. Renal ischemia-reperfusion leads to long term infiltration of activated and effector-memory T lymphocytes. Kidney Int (2009) 75(5):526–35. doi: 10.1038/ki.2008.602 PMC2676145 19092796

[224] Wan N Dai H Wang T Moore Y Zheng XX Dai Z. Bystander central memory but not effector memory Cd8+ T cells suppress allograft rejection. J Immunol (2008) 180(1):113–21. doi: 10.4049/jimmunol.180.1.113 18097010

[225] Madariaga ML Kreisel D Madsen JC. Organ-specific differences in achieving tolerance. Curr Opin Organ Transplant (2015) 20(4):392–9. doi: 10.1097/MOT.0000000000000206 PMC4523268 26147678

[226] Kawai T Cosimi AB Spitzer TR Tolkoff-Rubin N Suthanthiran M Saidman SL. Hla-mismatched renal transplantation without maintenance immunosuppression. N Engl J Med (2008) 358(4):353–61. doi: 10.1056/NEJMoa071074 PMC2819046 18216355

[227] Gaynor JJ Ciancio G Guerra G Sageshima J Hanson L Roth D. Graft failure due to noncompliance among 628 kidney transplant recipients with long-term follow-up: A single-center observational study. Transplantation (2014) 97(9):925–33. doi: 10.1097/01.TP.0000438199.76531.4a 24445926

[228] Dobbels F De Geest S van Cleemput J Droogne W Vanhaecke J. Effect of late medication non-compliance on outcome after heart transplantation: A 5-year follow-up. J Heart Lung Transplant (2004) 23(11):1245–51. doi: 10.1016/j.healun.2003.09.016 15539122

[229] Drick N Seeliger B Fuge J Tudorache I Greer M Welte T. Self-reported non-adherence to immunosuppressive medication in adult lung transplant recipients-a single-center cross-sectional study. Clin Transplant (2018) 32(4):e13214. doi: 10.1111/ctr.13214 29380445

[230] Shi YX Liu CX Liu F Zhang HM Yu MM Jin YH. Efficacy of adherence-enhancing interventions for immunosuppressive therapy in solid organ transplant recipients: A systematic review and meta-analysis based on randomized controlled trials. Front Pharmacol (2020) 11:578887. doi: 10.3389/fphar.2020.578887 33192520 PMC7606769

